# Variations in cardiovascular disease under-diagnosis in England: national cross-sectional spatial analysis

**DOI:** 10.1186/1471-2261-11-12

**Published:** 2011-03-17

**Authors:** Michael Soljak, Edgar Samarasundera, Tejal Indulkar, Hannah Walford, Azeem Majeed

**Affiliations:** 1 Department of Primary Care & Public Health, Imperial College London, 3rd Floor, Reynolds Building, Charing Cross Campus, St Dunstan's Road, London W6 8RP, UK; 2 Eastern Region Public Health Observatory, IPH, University Forvie Site, Robinson Way, Cambridge, CB2 0SR, UK

## Abstract

**Background:**

There is under-diagnosis of cardiovascular disease (CVD) in the English population, despite financial incentives to encourage general practices to register new cases. We compared the modelled (expected) and diagnosed (observed) prevalence of three cardiovascular conditions- coronary heart disease (CHD), hypertension and stroke- at local level, their geographical variation, and population and healthcare predictors which might influence diagnosis.

**Methods:**

Cross-sectional observational study in all English local authorities (351) and general practices (8,372) comparing model-based expected prevalence with diagnosed prevalence on practice disease registers. Spatial analyses were used to identify geographic clusters and variation in regression relationships.

**Results:**

A total of 9,682,176 patients were on practice CHD, stroke and transient ischaemic attack, and hypertension registers. There was wide spatial variation in observed: expected prevalence ratios for all three diseases, with less than five per cent of expected cases diagnosed in some areas. London and the surrounding area showed statistically significant discrepancies in observed: expected prevalence ratios, with observed prevalence much lower than the epidemiological models predicted. The addition of general practitioner supply as a variable yielded stronger regression results for all three conditions.

**Conclusions:**

Despite almost universal access to free primary healthcare, there may be significant and highly variable under-diagnosis of CVD across England, which can be partially explained by persistent inequity in GP supply. Disease management studies should consider the possible impact of under-diagnosis on population health outcomes. Compared to classical regression modelling, spatial analytic techniques can provide additional information on risk factors for under-diagnosis, and can suggest where healthcare resources may be most needed.

## Background

Geographic variation in the incidence and prevalence of CVD is a well-known phenomenon in population surveys. Such variations in both CHD and stroke incidence and prevalence are largely explained by area- and person-specific factors such as population socio-economic composition, demographic structure and ethnic diversity [1-4], mediated through established CVD risk factors [5]. In the case of England, this is exemplified at a regional level by a North-South gradient in prevalence and outcomes, with higher prevalences in the North [6].

From a health services perspective, it is important to ensure that as high a proportion as possible of actual cases have been diagnosed and well-managed when secondary prevention is known to be effective, as is the case for CVD [7]. Under-diagnosis and/or under-treatment of CVD has been found in some previous United Kingdom (UK) studies [8,9], but it was not possible to differentiate these causes. The recent availability of registered prevalence, performance and outcome data through the Quality & Outcomes Framework (QOF) pay-for-performance programme, in which almost all UK general practices participate, has provided accurate counts of diagnosed disease prevalence, and assurance that identified CVD is being increasingly well-managed. For example, in 2008-9, achievement by English general practices of all the 89 QOF points available for CHD management was 99.1 per cent [10]. The QOF also provides a financial reward for registering new cases of disease.

However, the extent of under-diagnosis may adversely affect population CVD outcomes. Epidemiologic models can be used to provide estimates of expected prevalence for small populations, using local data for known risk factors. Such models can be used for:

• targeting case-finding initiatives, by comparing diagnosed/observed and expected prevalence

• supporting needs assessment

• strategic planning

• underpinning commissioning of health services by providing denominators

• health equity audit

• supporting resource allocation [11].

A recent study which compared observed (QOF-registered) and expected prevalences of CHD and hypertension suggested that under-diagnosis may vary geographically [12]. However, the CHD model used by the authors was derived from the General Practice Research Database, resulting in a lack of independence between the observed and predicted datasets. Several disease prevalence models are now available on the Association of Public Health Observatories' (APHO) website [13]. These provide independently-derived prevalence estimates for resident populations of English local authority (LA), primary care trust (PCT), and practice populations, which can be compared with QOF disease registers [10]. Using these models, we investigated the observed and expected prevalence of three cardiovascular conditions (CHD, hypertension and stroke) at LA level to identify unmet population health care needs, their geographical variation, and population and healthcare predictors which might influence diagnosis.

## Methods

### Data sources: registered prevalence and primary care supply

Counts of general practice-registered, i.e. observed prevalence of CHD, stroke and hypertension in April 2007 were obtained from English general practice disease registers, produced for the purposes of incentivizing practices for achievement of QOF treatment targets. These patients are clinically confirmed cases of CVD who are receiving regular follow-up for their disease. QOF prevalence rates are based on total populations registered with practices, but to compare observed prevalence with expected prevalence estimated from resident population-based contextual data from the Census and other sources e.g. deprivation and proportion ethnic minority population, we derived residence-based QOF prevalence estimates for LAs using a lookup table - a pooled extract of England practice registers - from the National Strategic Tracing Service (now Personal Demographics Service) which apportioned practice populations to LA areas as at January 2006, by providing the exact number of practice population resident in each LA [14].

We apportioned counts of CVD patients registered by practices to LAs, in accordance with the proportion of each practice population resident in that area, which assumed that CVD prevalence was geographically uniform across a practice population (the average practice population is only about 6,400). We divided the aggregated count of CVD patients in each LA by total mid-2006 LA population estimates to give estimated crude prevalence, as used for QOF prevalence. Where less than 50 patients fell into an LA, the numbers were excluded from the look-up process. Three LAs could not be mapped due to discrepancies between QOF and NSTS datasets. In order to investigate the effect of healthcare supply upon diagnosis levels we included a measure of general practitioner availability in the form of the number of general practitioners (GPs) per thousand LA population, calculated in the same way [15].

### Data sources: expected/estimated prevalence

Expected prevalence for each LA was obtained from the APHO epidemiologic models, which are based on the socio-demographic and behavioural characteristics of respondents with the respective conditions in the Health Survey for England (HSfE). To produce the models, HSfE data for the years 2003-4 was pooled (sample size 21,233) to increase the cases of diseases. Surveys over this period included boosts for ethnic minority and older people, and focused on CVD and its risk factors. Of the respondents included, 14,574 (68.6%) were of White ethnicity, 308 were Mixed (1.5%), 1,991 (9.4%) were Black or Black British, and 3,725 (17.5%) Asian or Asian British. The outcome variables were patient-reported doctor-diagnosed CHD and stroke, and for hypertension, normotensive-treated, hypertensive-treated but uncontrolled, and hypertensive-untreated groups; i.e. a combination of patient-reported and objectively-measured variables. Patient reports of doctor-diagnosed CHD and stroke have been extensively validated elsewhere [16-20]. For example, in the British Regional Heart Study, 80 per cent of men with a GP record of angina reported their diagnosis, and 70 per cent of men who reported an angina diagnosis had confirmation of this from the record review. The prevalence of diagnosed angina in these older men was 10.1 per cent according to self-reported history and 8.9 per cent according to GP record review [16]. At that time (1999) some cases, such as newly-registered patients, may not have had their diagnosis clearly recorded in GP records.

Ordinary least-squares (OLS) logistic regression models were fitted and explanatory variables for each disease outcome identified by reverse stepwise selection.

The baseline odds of each disease were obtained directly from the HSfE dataset. The strength of association between each explanatory variable and disease caseness was then used to calculate the relative odds, which were applied to the baseline odds to derive the prevalence estimates for each sub-group of risk factors. The variables which can be included in each local model are limited by the availability of local data for them from Census and other national sources. The core model variables are ten-year age band, gender (male and female), ethnicity (Asian/Asian British, Black/Black British, White, Mixed and Other including Chinese) and deprivation (based on Index of Multiple Deprivation 2004 scores) [21]. In the case of CHD and stroke, smoking prevalence is also included, and the stroke model does not include ethnicity. The models use 2006 mid-year quinary age-band population estimates by ethnic group from the UK Office for National Statistics (ONS), which were summed to 10-year age bands to match the model [22]. LAs are stratified into deprivation score bands based on cut-offs of Lower Super Output Area quintiles - the ONS categories used in the HSfE. Internal validation included using the models to predict the response for each subject in the source data, and area under the receiver operating characteristics (AUROC) curve. AUROC curve values were 0.834, 0.844, and 0.807 for the stroke, CHD and hypertension models respectively. External validation showed that prevalence gradients derived from the models - for example with age and smoking status - agree well with published results from population-based studies.

In the case of the CHD and stroke models, smoking status is also included. Local smoking prevalence estimates are not available from the HSfE because of small sample sizes, so the CHD model uses synthetic estimates from the Neighbourhood Statistics website, which are for the period 2003-2005 [23]. Model assumptions include that the proportion of smokers, ex-smokers and never-smokers is uniform across ethnic categories and that the proportion of ex-smokers in each age-sex group is constant across areas. Sensitivity analysis has shown that varying the smoking prevalence has a very small effect on prevalence. Further technical details of the models are available in additional files 1 (CHD), 2 (hypertension) and 3 (stroke) in the web appendix, and also on the APHO website [13].

### Spatial analyses

Observed: expected prevalence ratios for LAs were calculated in Excel 2007 and mapped using the geographic information systems package ArcGIS 9. Two exploratory spatial data analysis methods commonly used in geographical studies were used to investigate patterns in O:E relationships, Local Moran's I (LMI) analysis and geographically weighted regression (GWR). The LMI technique is used to identify geographic clusters and outliers in data by testing for randomness in spatial distribution across a dataset, localities with significance scores (Z scores) greater than two standard deviations being considered to be either clusters or outliers [24]. Strongly positive Z scores indicate statistically significant similar values in close geographic proximity hence the presence of a cluster; a strongly negative Z score demonstrates a locality with a significantly dissimilar value in relation to its neighbouring localities thus indicating an outlier.

GWR is a form of spatial statistics which disaggregates geographic data into spatial blocks using a probability distribution kernel, which moves from location to location across the dataset to test for geographic variation in regression relationships. In situations where there is geographic variation in the strength of a regression relationship, a phenomenon referred to as spatial non-stationarity, the use of GWR will improve model goodness-of-fit to data, expressed as the trade-off between statistical predictor bias (linked to R-squared values) and variance (linked to degrees of freedom). In comparison to a classical model GWR will produce higher correlation coefficients, lower residuals and higher degrees of freedom than traditional ordinary least squares (OLS) regression [25,26].

In this paper, we used GWR to assess whether a linear regression relationship between observed and expected prevalence existed and if so whether it varied in strength over space, the purpose of which should be viewed as distinct from that of mapping observed to expected ratios, as the latter aims to measure equality between two variables rather than to assess predictability of an association. Both OLS and GWR models were run in the software package GWR 3 to test for spatial non-stationarity. The optimal bandwidth for the kernel was estimated using the Akaike Information Criterion [27]. Two rounds of regression were performed, the first a univariate regression involving expected prevalence as the independent variable and observed prevalence as the dependent variable; the second bivariate including whole-time equivalent GP supply as an additional variable.

Our research conformed to the Helsinki Declaration http://www.wma.net/en/30publications/10policies/b3/, and to local legislation. It did not require ethical approval or patient consent as it is a secondary analysis of publicly-available data.

## Results

### Observed/diagnosed prevalence of CVD

Observed (O) and expected (E) prevalence summary statistics are shown in Table 1. Total English population was used as the denominator for consistency with standard reporting of QOF prevalence. The mean prevalence of QOF-diagnosed CHD in LAs was 2.57% (95% CIs 2.45-2.69), and the expected prevalence was 4.52% (95% CIs 4.42-4.61), giving an O:E ratio of 0.57 i.e. about 60 per cent of expected cases are diagnosed. Although the prevalence of stroke is less than half that of CHD, the O:E ratio is very similar. The expected prevalence of hypertension is much higher (about 24% of over 16s), and the O:E ratio is only 0.37 i.e. less than 40% of cases may be diagnosed. There was wide variation in the O:E ratio between LAs, with less than five per cent of expected cases diagnosed in some areas for all three diseases.

**Table 1:** observed (GP-registered) and expected/modelled prevalence summary statistics

	Coronary Heart Disease			Hypertension			Stroke		
	**Observed Prevalence**	**Expected Prevalence**	**O:E Ratio**	**Observed Prevalence**	**Expected Prevalence**	**Observed: Expected Ratio**	**Observed Prevalence**	**Expected Prevalence**	**O:E Ratio**

**Number**	1,299,601	2,286,597		4,530,369	12,356,995		588,500	1,009,144	

**Prevalence rate/Percent (95% CIs)^1^**	2.57% (2.45%-2.69%)	4.52% (4.42%-4.61%)	0.57 (0.55-0.59)	8.95% (8.59%-9.32%)	24.42% (24.13%-24.72%)	0.37 (0.35-0.38)	1.16% (1.11%-1.22%)	1.99% (1.96-2.03%)	0.58 (0.56-0.61)

**Median**	2.40%	4.48%	0.55	8.77%	24.77%	0.36	1.11%	1.99%	0.57

**Maximum**	5.47%	7.78%	1.10	16.62%	35.17%	0.63	2.48%	3.26%	1.08

**Minimum**	0.14%	2.61%	0.04	0.57%	17.71%	0.02	0.06%	1.24%	0.03

**1st quartile**	1.56%	3.89%	0.38	5.92%	23.06%	0.25	0.74%	1.77%	0.40

**3rd quartile**	3.31%	5.10%	0.69	11.10%	26.75%	0.43	1.49%	2.23%	0.73

**Interquartile range**	1.75%	1.22%	0.32	5.18%	3.69%	0.18	0.75%	0.47%	0.33

**Standard deviation**	0.011	0.009	0.21	0.035	0.028	0.13	0.005	0.004	0.22

^1^The England population denominator used for all three diseases was 50,598,200

### Spatial analysis

**Figure 1:**
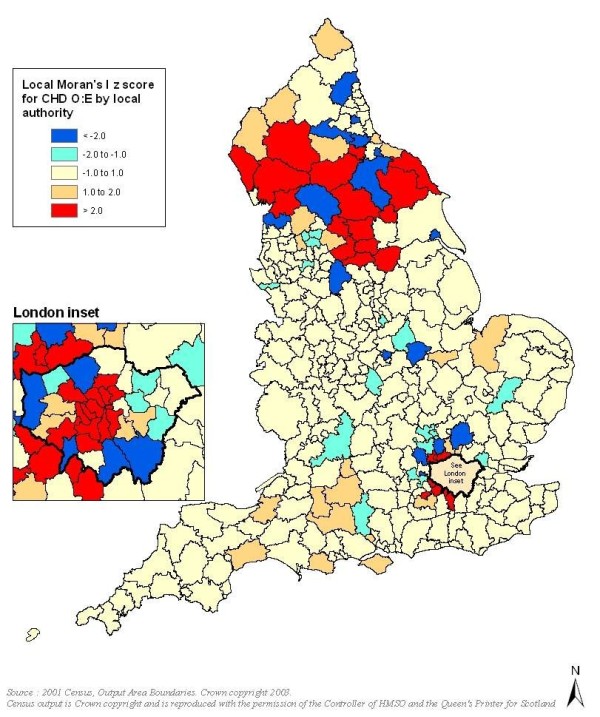
**Clusters and outliers in observed to expected ratios for CHD (red indicates clusters and blue indicates outliers)**.

**Figure 2:**
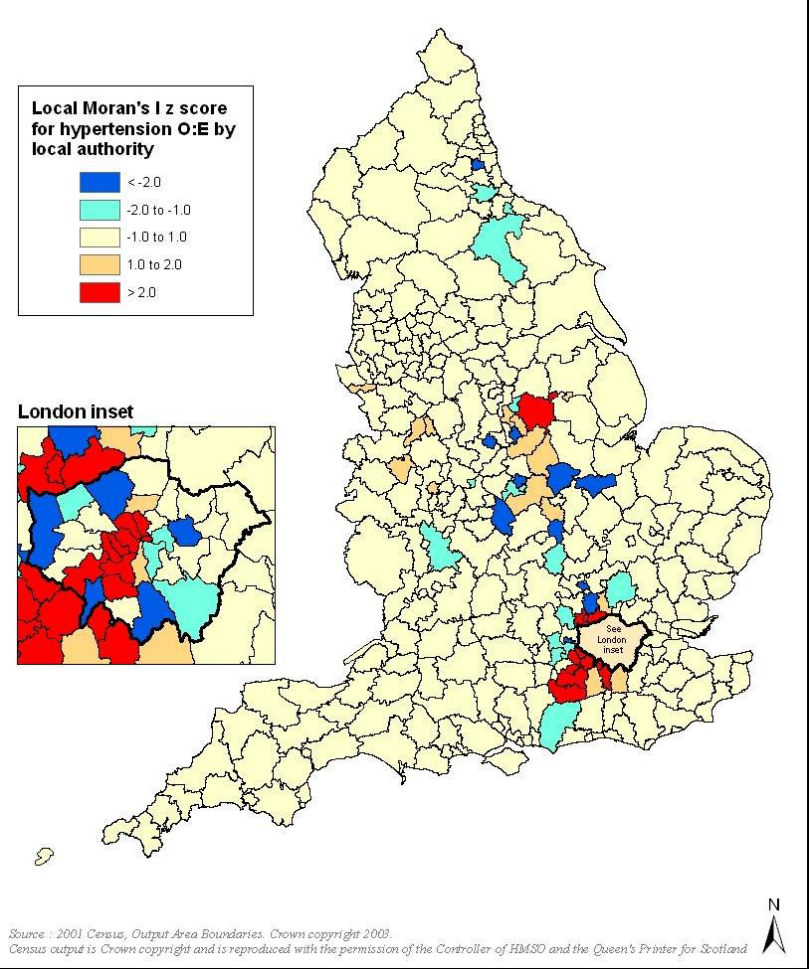
**Clusters and outliers in observed to expected ratios for hypertension (red indicates clusters and blue indicates outliers)**.

**Figure 3:**
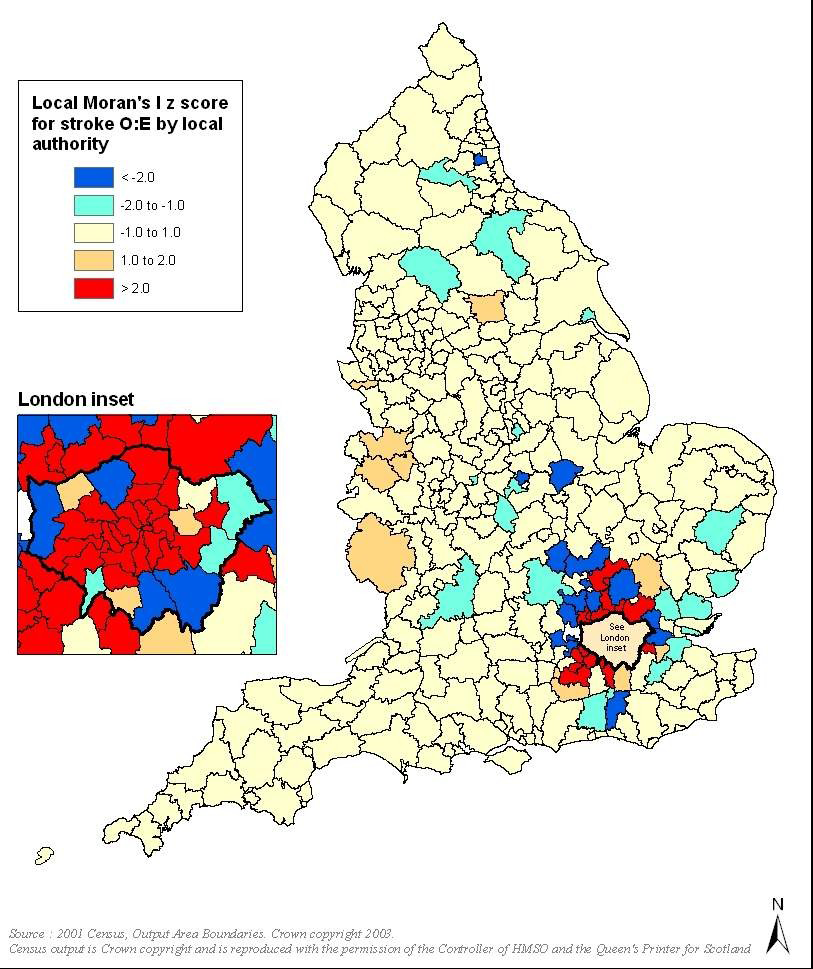
**Clusters and outliers in observed to expected ratios for stroke (red indicates clusters and blue indicates outliers)**.

Mapping of observed and expected prevalence demonstrated that observed and predicted prevalence for both diseases showed strong north-south and southwest-southeast gradients, with the southeast generally showing lowest disease levels, especially in the case of expected prevalence. However, within these geographical trends in both QOF and modelled prevalence, mapping of O:E ratios demonstrated spatial variation between neighbouring LAs. LMI Z scores indicated the presence of some statistically significant clusters and outliers in O:E ratios (Figure 1, Figure 2 and Figure 3).

For all three conditions, particularly CHD and stroke, London and much of its hinterland showed statistically significant discrepancies in O:E ratios, with observed prevalence much lower than the epidemiological models predicted. For CHD there were also significant clusters and outliers in parts of northern England, especially in Cumbria and Yorkshire, though unlike for the London area the clusters typically related to O:E ratios tending towards unity.

Further geographic analysis with GWR revealed significant spatial variation in the O:E relationship for CHD, hypertension and stroke, with GWR describing the dataset more accurately than traditional OLS regression. The linearity of the relationship between observed and expected prevalence was weakest for hypertension with the lowest overall regression coefficients and highest residual sum of squares for observed against expected prevalence; stronger associations were observed for CHD and stroke.

Even maximum coefficient values for hypertension, found along the south and east coasts, were less than 0.26; coefficients were lowest in the north and a pronounced north-south gradient was observed. The pattern for stroke was somewhat more complex with coefficients typically less than 0.21 for much of the northern parts of the country and the Midlands, yet with parts of Kent and the Sussex Counties having coefficients up to 0.69. CHD followed a similar geographic structure to stroke, with values of less than 0.08 for much of the Midlands and north, yet rising to 0.66 in eastern and southern coastal areas. There were no statistically significant patterns in Cook's D and standardised residual results for any of the three conditions examined. However, although O:E ratios tended towards 1 in the north and declined further south, the GWR results showed that the predictability of the relationship between observed and expected prevalence decreased with an increasing latitude. The addition of GP supply as a variable yielded stronger regression results for all three conditions (Table 2).

**Table 2:** comparison of classical regression and GWR results

CHD	Classical regression		GWR	
	
	O against E	O = E+GP	O against E	O = E+GP
Residual sum of squares	0.032	0.026	0.026	0.017

Standard deviation	0.010	0.009	0.009	0.007

Akaike Information Criterion	-2260.43	-2336.25	-2285.75	-2393.81

Correlation coefficient	0.299	0.439	0.427	0.637

Adjusted correlation coefficient	0.295	0.434	0.387	0.585

Sum of squares	0.0	0.0	0.0	0.0

Degrees of freedom	2.00	3.00	328.09	306.86

**Hypertension**	**Classical regression**		**GWR**	
	
	**O against E**	**O = E+GP**	**O against E**	**O = E+GP**

Residual sum of squares	0.374	0.250	0.362	0.241

Standard deviation	0.033	0.027	0.032	0.026

Akaike Information Criterion	-1400.41	-1539.57	-1403.50	-1543.67

Correlation coefficient	0.121	0.412	0.150	0.432

Adjusted correlation coefficient	0.116	0.407	0.134	0.421

Sum of squares	0.4	0.2	0.4	0.2

Degrees of freedom	2.00	3.00	344.87	344.04

**Stroke**	**Classical regression**		**GWR**	
	
	**O against E**	**O = E+GP**	**O against E**	**O = E+GP**

Residual sum of squares	0.007	0.006	0.005	0.003

Standard deviation	0.004	0.004	0.004	0.003

Akaike Information Criterion	-2807.25	-2873.16	-2838.92	-2932.93

Correlation coefficient	0.262	0.392	0.422	0.621

Adjusted correlation coefficient	0.258	0.387	0.374	0.561

	**Classical regression**		**GWR**	
	
	**O against E**	**O = E+GP**	**O against E**	**O = E+GP**

Sum of squares	0.0	0.0	0.0	0.0

Degrees of freedom	2.00	3.00	324.37	302.87

O against E: ratio of observed against expected prevalence

O = E+GP: inclusion of GP supply as an additional independent variable

## Discussion

### Main findings

The findings presented here indicate that despite almost universal access to free primary healthcare services, and a significant financial incentive for these services to find and register new cases on their computer systems, there may be significant under-diagnosis of all three CVD conditions examined in this study in many areas in England. The difference between expected and recorded disease is most marked for hypertension, with strikingly wide variation in diagnosis levels between areas. Hypertension and stroke showed similar geographic variation to CHD, but without the clusters/outliers for northern England; additionally the contrast between the ratios for London and LAs immediately to the north was more pronounced for stroke.

An obvious question is: why was there such a discrepancy between expected and practice-registered disease prevalence, when the model-based CHD and stroke prevalence estimates were based on patient reports of doctor-diagnosed disease? Reasons why practice computer systems may under-record cases are likely to include inadequate searching of practice records (which are now in the UK mainly electronic) for previous diagnoses or CVD-related prescriptions [28,29], lack of linkage of practice and hospital records (practice still have to enter codes for hospital admissions manually), and high population mobility in urban areas: data for new patients is often still entered into practice systems manually and previous diagnoses may not be added for some time [30]. The QOF does not appear to have resulted in substantial improvement in recording. The crude QOF CHD prevalence was 3.44% in 2006-7, the year after QOF was implemented, but was 3.54% in 2009/10 - a minimal increase. In the case of hypertension, for which the modelled estimates used a combination of doctor-diagnosed disease and blood pressure measurements, the gap is greatest in younger and middle age groups, when males in particular are less likely to be seen by their practices [31]. In contrast, diagnosis appears to be more complete in the north of England.

From a spatial analytic perspective, there are significant geographic discrepancies between QOF prevalence and modelled prevalence for all three conditions in much of London and its hinterland. The inclusion of a measure of GP supply in the GWR analyses suggests that, despite needs-based healthcare resource allocation in the UK, persistent differences in availability of primary care services is an important limiting factor in diagnosis. An analysis of Gini coefficients to measure geographical equity in GPs per 100,000 population in England and Scotland showed that equity in England rose between 1974 and 1994, but then decreased, and in 2006 it was below the 1974 level [32].

The results also provide some contrast to a study investigating revascularisation rates in males, which found that even after adjusting for the higher CHD burden in the north of the country related to socio-demographic composition, the likelihood of receiving surgery during the 1990s decreased outside of southern England, suggesting a geographic imbalance in the provision of tertiary cardiology care [33]. Considerable efforts have been made subsequently to ensure that access to tertiary care is more equitable. However our analysis suggests that from a primary care perspective, it is London and the surrounding area which would benefit most from increased resource allocation, although GP supply itself seems to be only one influential factor affecting diagnosis levels. Further work will need to investigate the effectiveness and yield of strategies for CVD case-finding by practices, and to validate the predictions of the prevalence models at practice level.

### Strengths and limitations of the study

Strengths of the study include the use of new data from practice disease registers and recently-developed geographical analytical techniques and software. Limitations include the fact that modelling was carried out at LA level, which may conceal much wider variation at lower levels. The spatial scale of the analyses includes many LAs which are highly heterogeneous in socio-demographic composition, so the study may have missed significant small area variation; this is especially likely in northern areas where regression associations were weakest. There is imprecision in the model prevalence estimates, for example, because of the size of the Health Survey sample we used, especially in subgroups with small numbers of cases - the precision of the estimates is also affected by the prevalence of the disease outcomes. Model restrictions include that the proportion of smokers, ex-smokers and never-smokers is uniform across ethnic categories, when, for example, smoking prevalence varied from 16.4% in the Black/Black British sample population to 24.1% in the White sample population. Smoking prevalence is itself a modelled estimate, and ex-smoking prevalence is assumed to be the same nationwide. In addition the number of variables in the local prevalence models is constrained by the availability of local data on risk factors, so that it is not possible to include other well-established CVD risk factors as variables. Some of the local risk factor data relies on estimates based on 2001 Census data, although this will be improved by, for example, more accurate and timely data on smoking prevalence from the new ONS Integrated Household Survey. However we were reassured by the results of model validation, e.g. ROC curves. In addition, comparing model predictions to QOF registrations is also a form of external validation. For example, QOF registered counts of CHD were greater than expected counts in only three of 352 LAs, and in these only by small amounts. If the models were very imprecise, much more under-estimation would be expected. Further model validation is needed, and is occurring as the models are being used to guide local case-finding initiatives.

## Conclusions

Despite the absence of barriers to primary healthcare, there is likely to be significant and highly variable under-diagnosis of CVD across England, which can be partially explained by persistent inequity in GP supply. However, the distinctive composition and dynamics of London's population probably adds complexities to the identification of CVD in that region. Studies of disease management should consider the impact of this "iceberg" of undiagnosed disease on hospital utilisation and population health outcomes. Spatial analytic techniques can provide additional information about geographical variation compared to classical regression modelling, and can suggest where more healthcare resources may be most needed.

## Data sharing

Datasets and statistical code for GWR are available from the corresponding author at m.soljak@imperial.ac.uk

## Competing interests

The authors declare that they have no competing interests.

## Authors' contributions

MS and HW developed the prevalence models. MS conceived and planned the study, and wrote the first draft of the paper. ES and TI undertook the spatial analysis, and all authors were involved in the interpretation of the data. All authors revised it critically for important intellectual content and approved the final version.

## Pre-publication history

The pre-publication history for this paper can be accessed here:

http://www.biomedcentral.com/1471-2261/11/12/prepub

## Supplementary Material

Additional file 1 **CHD prevalence modelling briefing document v5**. This document describes how the CHD prevalence model was developed from Health Survey for England data and how the model was applied to local population data. Click here for file

Additional file 2 **Hypertension prevalence modelling briefing document v2**. This document describes how the hypertension prevalence model was developed from Health Survey for England data and how the model was applied to local population data. Click here for file

Additional file 3 **Stroke prevalence modelling briefing document v2**. This document describes how the hypertension prevalence model was developed from Health Survey for England data and how the model was applied to local population data. Click here for file

## References

[B1] Cox AM McKevitt C Rudd AG Wolfe CD Socioeconomic status and stroke Lancet Neurol 2006 5 2 181 188 10.1016/S1474-4422(06)70351-9 16426994

[B2] Hajat C Tilling K Stewart JA Lemic-Stojcevic N Wolfe CDA Ethnic Differences in Risk Factors for Ischemic Stroke: A European Case-Control Study Stroke 2004 35 7 1562 1567 10.1161/01.STR.0000131903.04708.b8 15192251

[B3] Hemingway H Langenberg C Damant J Frost C Pyorala K Barrett-Connor E Prevalence of Angina in Women Versus Men: A Systematic Review and Meta-Analysis of International Variations Across 31 Countries Circulation 2008 117 12 1526 1536 10.1161/CIRCULATIONAHA.107.720953 18347213

[B4] McFadden E Luben R Wareham N Bingham S Khaw KT Social Class, Risk Factors, and Stroke Incidence in Men and Women. A Prospective Study in the European Prospective Investigation Into Cancer in Norfolk Cohort Stroke 2009 40 4 1070 1077 10.1161/STROKEAHA.108.533414 19228844

[B5] Morris RW Whincup PH Lampe FC Walker M Wannamethee SG Shaper AG Geographic variation in incidence of coronary heart disease in Britain: the contribution of established risk factors Heart 2001 86 3 277 283 10.1136/heart.86.3.277 11514478 PMC1729899

[B6] Doran T Drever F Whitehead M Health underachievement and overachievement in English local authorities J Epidemiol Comm Health 2006 60 8 686 693 10.1136/jech.2005.041889 PMC2588077 16840758

[B7] Clark AM Hartling L Vandermeer B McAlister FA Meta-Analysis: Secondary Prevention Programs for Patients with Coronary Artery Disease Ann Intern Med 2005 143 9 659 672 16263889 10.7326/0003-4819-143-9-200511010-00010

[B8] Ashworth M Lloyd D Smith RS Wagner A Rowlands G Social deprivation and statin prescribing: a cross-sectional analysis using data from the new UK general practitioner Quality and Outcomes Framework J Publ Health 2007 29 1 40 47 10.1093/pubmed/fdl068 17071815

[B9] Ward P Noyce P Leger A Are GP practice prescribing rates for coronary heart disease drugs equitable? A cross sectional analysis in four primary care trusts in England J Epidemiol Comm Health 2004 58 89 96 10.1136/jech.58.2.89 PMC1732682 14729882

[B10] The Information Centre Quality and Outcomes Framework (QOF) for April 2006 to March 2007, England: Numbers of patients on QOF disease registers, and unadjusted prevalence rates The Information Centre 2008 http://www.ic.nhs.uk/webfiles/QOF/2006-07/National%20QOF%20tables%202006-07%20-%20prevalence.xls [cited 2008 Apr. 25];

[B11] Soljak M Flowers J Closing the Gap: Using Prevalence Models for Long-term Conditions in the United Kingdom J Ambul Care Manage 2008 31 3 211 215 18574378 10.1097/01.JAC.0000324665.98777.b6

[B12] Campbell SM Reeves D Kontopantelis E Sibbald B Roland M Effects of Pay for Performance on the Quality of Primary Care in England N Engl J Med 2009 361 4 368 378 10.1056/NEJMsa0807651 19625717

[B13] Association of Public Health Observatories Browsing Disease prevalence models 2009 APHO http://www.apho.org.uk/resource/view.aspx?RID=48308 cited 10/3/2010.

[B14] Connecting for Health The Personal Demographics Service 2010 Connecting for Health http://www.connectingforhealth.nhs.uk/systemsandservices/demographics/pds cited 30/9/2010.

[B15] Information Centre for Health & Social Care NHS Staff 1996 - 2006 (General Practice) 2010 Information Centre for health & social care http://www.ic.nhs.uk/statistics-and-data-collections/workforce/nhs-staff-numbers/nhs-staff-1996--2006-general-practice cited 30/9/2010.

[B16] Lampe FC Walker M Lennon LT Whincup PH Ebrahim S Validity of a Self-reported History of Doctor-diagnosed Angina J Clin Epidemiol 1999 52 1 73 81 10.1016/S0895-4356(98)00146-2 9973076

[B17] Glymour MM Avendano M Can Self-Reported Strokes Be Used to Study Stroke Incidence and Risk Factors? Evidence From the Health and Retirement Study Stroke 2009 40 3 873 879 10.1161/STROKEAHA.108.529479 19150869

[B18] Kazumasa Y Ai I Hiroyasu I Manami I Shoichiro T Self-reported stroke and myocardial infarction had adequate sensitivity in a population-based prospective study Journal of clinical epidemiology 2009 62 6 667 673 10.1016/j.jclinepi.2008.07.016 19108984

[B19] O'Mahony PG Dobson R Rodgers H James OFW Thomson RG Validation of a Population Screening Questionnaire to Assess Prevalence of Stroke Stroke 1995 26 8 1334 1337 7631332 10.1161/01.str.26.8.1334

[B20] Walker MK Whincup PH Shaper AG Lennon LT Thomson AG Validation of Patient Recall of Doctor-diagnosed Heart Attack and Stroke: A Postal Questionnaire and Record Review Comparison Am J Epidemio 1998 148 4 355 361 10.1093/oxfordjournals.aje.a009653 9717879

[B21] Department of Communities & Local Government The English Indices of Deprivation 2004 (revised) 2008 DCLG http://www.communities.gov.uk/publications/communities/englishindices cited 18/7/2008.

[B22] Office for National Statistics Population Estimates by Ethnic Group (experimental) National Statistics Online 2007 http://www.statistics.gov.uk/statbase/Product.asp?vlnk=14238 cited 18/7/2008.

[B23] Office for National Statistics Model Based Estimate for Smoking in Healthy Lifestyle Behaviours: Model Based Estimates, 2003-2005 2007 Neighbourhood Statistics http://www.neighbourhood.statistics.gov.uk/dissemination/viewFullDataset.do?$ph=60_61_62&step=4&productId=969&instanceSelection=022951&timeId=242&containerAreaId=276706&startColumn=1&numberOfColumns=8&viewAction=parent

[B24] Anselin L Local indicators of spatial association: LISA Geog Anal 2009 27 93 115 10.1111/j.1538-4632.1995.tb00338.x

[B25] Brunsdon C Fotheringham S Charlton M Geographically weighted regression-modelling spatial non-stationarity J Royal Stat Soc: Series D (The Statistician) 1998 47 3 431 10.1111/1467-9884.00145

[B26] Fotheringham AS Brunsdon C Charlton M Geographically Weighted Regression: The Analysis of Spatially Varying Relationships 2002 Chichester: Wiley

[B27] Akaike H A new look at statistical model identification Automatic Control, IEEE Transact 1974 19 6 716 723 10.1109/TAC.1974.1100705

[B28] Gray J Majeed A Kerry S Rowlands G Identifying patients with ischaemic heart disease in general practice: cross sectional study of paper and computerised medical records BMJ 2000 321 7260 548 550 10.1136/bmj.321.7260.548 10968818 PMC27471

[B29] Donnan PT Dougall HT Sullivan FM Optimal strategies for identifying patients with myocardial infarction in general practice Fam Pract 2003 20 6 706 710 10.1093/fampra/cmg614 14701896

[B30] Millett C Zelenyanszki C Binysh K Lancaster J Majeed A Population mobility: characteristics of people registering with general practices Pub Health 2005 119 7 632 638 10.1016/j.puhe.2004.09.004 15885722

[B31] Craig R Mindell J Health Survey for England 2006: Volume 1 CVD and risk factors adults, obesity and risk factors children Information Centre for Health & Social Care 2008 http://www.ic.nhs.uk/webfiles/publications/HSE06/HSE%2006%20report%20VOL%201%20v2.pdf cited 26/11/2008.

[B32] Goddard M Gravelle H Hole A Marini G Where did all the GPs go? Increasing supply and geographical equity in England and Scotland J Health Serv Res Policy 2010 15 1 28 35 10.1258/jhsrp.2009.009003 19843638

[B33] Morris RW Whincup PH Papacosta O Walker M Thomson A Inequalities in coronary revascularisation during the 1990s: evidence from the British regional heart study Heart 2005 91 5 635 640 10.1136/hrt.2004.037507 15831650 PMC1768900

