# Supplementary material for: Variations in cardiovascular disease under-diagnosis in England: national cross-sectional spatial analysis
Source: BMC Cardiovasc Disord. 2011 Mar 17;11:12. doi: 10.1186/1471-2261-11-12 (PMC3070686; doi:10.1186/1471-2261-11-12)
Supplement: Additional file 1 — CHD prevalence modelling briefing document v5. This document describes how the CHD prevalence model was developed from Health Survey for England data and how the model was applied to local population data. [file 1471-2261-11-12-S1.PDF]

CHD Prevalence Modelling Briefing Document

Hannah Walford, ERPHO

Michael Soljak, Fabiana Gordon, Department of Primary Care and Social Medicine & Statistical Advisory Service, Imperial College London

November 2008

This briefing document explains how the CHD (Coronary Heart Disease) prevalence model has been developed and applied. It accompanies the release of CHD prevalence estimates in September 2008.

Contents

1 Background 3

2 Model Development 3

2.1 Background 3

2.1.1 Defining prevalence 3

2.1.2 Other prevalence surveys 5

2.1.3 Previous CHD prevalence modelling 7

2.2 Methods 7

2.2.1 Data sources 7

2.2.2 Model construction: data issues 8

2.2.3 Model construction: interactions between variables 9

2.2.4 Model construction: internal validation 10

2.2.5 Model construction: external validation 11

2.3 The Model 11

2.3.1 Local model 12

2.3.2 Complete model excluding treatment for hypertension 13

2.3.3 Complete model including treatment for hypertension 14

2.3.4 Validation: area under Receiver Operating Characteristics curve (AUROC) 16

2.3.5 Validation: model prediction 17

3 Application of the Model 18

3.1 Assumptions of the modelled estimates 18

3.2 Input data 18

3.2.1 Populations 18

3.2.2 Smoking status 19

3.2.3 Deprivation 21

4 References 21

1 Background

The disparity between CHD prevalence estimates from large surveys, in particular the Health Survey for England (HSfE), and the number of patients diagnosed with CHD and registered in QOF led to demand for a CHD prevalence model at PCT and Local Authority level that gives an accurate estimate of true prevalence. The Association of Public Health Observatories (APHO) published a simple prevalence model to support development of 2007-08 Local Delivery Plans (1). However, it was acknowledged that this was a crude model and APHO subsequently commissioned Michael Soljak of the Department of Primary Care and Social Medicine, Imperial College, London to develop a more robust model.

2 Model Development

Coronary heart disease (CHD) is a relatively common condition, for which there are a number of evidence-based treatments which are known to reduce mortality. The HSfE for 2006 estimates that, based on respondents' self-reports of doctor-diagnosed CHD, the prevalence is about 6.5 per cent in males aged 16+ and 4.0 per cent in females aged 16+ (1), and this increases markedly with age. This prevalence has remained static over the last ten years. However the Quality and Outcomes Framework (QOF) of the GP Contract, covering over 8,000 practices and 53 million patients, shows a GP-registered unadjusted prevalence of only 3.5 per cent (2) (but note that unadjusted prevalence rates show these registers as a percentage of the total practice list size i.e. all ages).

2.1 Background

2.1.1 Defining prevalence

There are differences between various methods of estimating CHD prevalence. The positive predictive value of questionnaire responses such as those used in HSfE to define CHD may be sub-optimal in comparison with clinical diagnosis. Conversely, reliance on a medical diagnosis may underestimate prevalence, as patients with unrecognised angina or very mild symptoms may not attend (or be correctly identified by) their GP.

A Belgian analysis of the records of four large Belgian epidemiological studies during the past 30 years compared clinical and electrocardiographic (ECG) findings (see Table 1) (3). Q wave patterns, ST segment depression or elevation, T wave inversion or flattening, and left bundle branch block are often seen as indications of silent myocardial ischaemia. The occurrence of ischaemia-like findings on the ECG was comparable between men and women (9.0% *v* 9.8%). The results from this and other studies consistently show that ischaemia-like ECG changes are associated with an approximately twofold increased risk of dying of CHD.

Table 1   Prevalences of coronary heart disease and ECG findings in 25-74 year old men and women (4)

	Sex	25-34 years	35-44 years	45-5 years	55-64 years	65-74 years	25-74 years*	Odds ratios (men v women) (95% CI)
Angina pectoris	M	2.50%	3.10%	4.90%	7.90%	13.10%	5.00%	0.51 (0.46 to 0.56) for age < 55 years,
	F	4.00%	6.70%	8.50%	8.40%	11.90%	6.00%	1.00 (0.85 to 1.18) for age>55 years
History of acute MI	M	0.00%	0.60%	2.40%	6.30%	12.80%	3.60%	2.66 (2.20 to 3.22)
	F	0.10%	0.20%	0.60%	3.00%	5.60%	1.50%	
Minnesota codes $I_{1,2}$	M	0.40%	0.90%	1.70%	3.00%	5.00%	1.80%	2.02 (1.63 to 2.48)
	F	0.00%	0.40%	0.80%	1.60%	3.30%	0.90%	
Coronary heart disease	M	2.90%	4.30%	7.80%	13.60%	22.70%	8.30%	0.70 (0.64 to 0.77) for age < 55 years
	F	4.00%	7.10%	9.60%	11.40%	17.60%	7.60%	1.22 (1.06 to 1.39) for age > 55 years
Major ECG findings	M	1.60%	2.80%	4.80%	9.30%	19.00%	6.00%	1.42 (1.27 to 1.57)
	F	0.80%	2.00%	3.40%	6.70%	14.60%	4.30%	
Minor ECG findings	M	3.40%	5.50%	9.20%	16.60%	29.40%	10.40%	1.13 (1.05 to 1.22)
	F	3.50%	3.90%	8.30%	16.10%	29.50%	9.50%	

In the British Regional Heart Study (BRHS), there was considerable overlap of questionnaire and ECG evidence of CHD, and high agreement between self-report and medical record for diagnosed CHD: for example, 80% of men with a GP record of angina reported their diagnosis, and 70% of men who reported an angina diagnosis had confirmation of this from the record review (4-6). The prevalence of diagnosed angina in 1992 in these older men was 10.1% according to self-reported history and 8.9% according to GP record review.

Nevertheless, more than half of those in the BRHS with possible myocardial infarction (MI) combined with angina had no resting electrocardiographic evidence of CHD, and half of those with definite myocardial infarction on electrocardiogram had no history of chest pain at any time (7;8). Only half of those with a definite MI on an electrocardiogram could recall a medical diagnosis of CHD (9). Even in severe (grade 2) angina 40% could not recall being told that they had heart disease. Overall, only one in five of those regarded as having CHD was able to recall such a diagnosis having been made by a doctor, and these were likely to be those most severely affected.

However there was substantial agreement between self-report and GP record of angina. The BRHS subsequently combined two questionnaire-based definitions to define prevalence: either *current angina symptoms*, which were defined as a positive response to standard World Health Organization (Rose) questionnaires (overall prevalence 11.1%); or *history of diagnosed CHD* was defined as subject recall of ever having had a doctor's diagnosis of either angina or heart attack (overall prevalence also 11.1%) (10).

On the other hand the HSfE uses only the latter questionnaire definition in its prevalence estimates. Trends in prevalence are shown in Table 2. These are not dissimilar to the BRHS given the latter only included males aged 40-59.

Table 2: Percentage prevalence of IHD, by HSfE survey year, age and sex (11)

Age	1994		1998		2003 Unweighted		2006 Unweighted		2003' Weighted		2006 Weighted	
	M	F	M	F	M	F	M	F	M	F	M	F
16-24	-	0.2	0.1	-	-	0.2	0.2	0.1	-	0.3	0.1	0.1
25-34	0.3	0.1	0.4	0.3	-	-	0.2	0.2	-	-	0.2	0.1
35-44	0.5	0.3	0.9	0.6	0.9	0.4	0.7	0.3	1.0	0.5	0.6	0.3
45-54	3.0	2.3	4.3	1.8	3.5	2.0	3.7	1.3	3.4	1.9	3.6	1.3
55-64	10.3	5.9	13.6	6.3	11.1	5.9	10.7	3.4	11.1	5.8	10.6	3.5
65-74	21.0	10.5	20.2	12.5	21.5	9.7	20.6	10.2	21.6	9.7	20.8	10.0
75+	22.7	15.9	23.4	18.4	26.4	18.4	28.5	19.3	26.5	18.1	28.4	19.3
All	6.0	4.1	7.1	4.6	7.4	4.5	6.2	3.0	6.4	4.1	6.5	4.0

2.1.2 Other prevalence surveys

While CHD mortality has greatly declined in the last four decades, the use of age-adjusted rates to describe CHD mortality obscures the fact that the decline largely represents the postponement of CHD deaths until older age. In fact, the overall burden of CHD is increasing in parallel with the increase in life expectancy. As the burden of prevalent CHD is increasing, identifying persons with CHD, measuring its incidence and outcome and how these vary over time and across populations is essential to understand the determinants of the trends in CHD. This in turn is crucial to define the relative contributions of risk factor reduction and therapeutic improvements, which is necessary to design effective interventions to reduce CHD.

We undertook a literature search for recent (post-1996) CHD prevalence surveys. We included only articles in English, which covered groups represented in the UK population in significant numbers. We also excluded surveys which covered CHD risk factor prevalences, or which sampled only sub-populations e.g. those with diabetes.

Community surveillance is a comprehensive approach designed to track disease at the community level, and is less costly and more efficient than cohort studies. In the USA, several community surveillance studies have reported on temporal trends in CHD prevalence e.g. the Atherosclerosis Risk in Communities (ARIC) study, the Minnesota Heart Survey, the Olmsted County Study, and the Worcester Heart Attack Study (12). In an analysis of US National Health & Nutrition Survey data on participants aged ≥ 40 years who attended the medical examination, the age-adjusted prevalence of angina pectoris, self-reported myocardial infarction, and ECG-defined myocardial infarction were 5.8% of 9255, 6.7% of 9250, and 3.0% of 8206 participants, respectively (13). The age-adjusted prevalence of coronary heart

disease defined by the presence of any of these conditions was 13.9% among men and 10.1% among women. These studies suggested that in the US medical care of clinical CHD was the main contributor to the mortality decline (14).

Outside the USA, the World Health Organization (WHO) MONICA (Multinational MONItoring of trends and determinants in CArdiovascular disease) Project was established in the early 1980s to monitor trends in cardiovascular diseases and to relate these to risk factor changes. Its central goal was to explain the trends in cardiovascular disease mortality observed from the 1970s. There were 32 MONICA centres in 21 countries. In these populations, the decline in coronary disease mortality is mostly related to the decline in CHD events, thereby pointing to primary prevention as the main source (15). However the study populations excluded over 65s in whom most CHD occurs.

In a survey of a rural Indian population, CHD was diagnosed on basis of past documentation, response to WHO-Rose questionnaire, or changes in ECG. The prevalence of CHD (clinical + ECG criteria) was 3.4% in males and 3.7% in females. According to ECG criteria only, it was 2.8% in males and 3.3% in females and according to Q-waves only, it was 1.6% in males and 0.9% in females (16). In a Finnish population survey Ahto et al found the prevalence of angina symptoms was 9.1% among men and 4.9% among women aged 64-97 (17). Ischaemic ECG findings were common: 32.9% of men and 39.3% of women had such changes. An international systematic review and meta-analysis found that angina prevalence varied widely across populations, from 0.73% to 14.4% (population weighted mean 6.7%) in women and from 0.76% to 15.1% (population weighted mean 5.7%) in men (18).

In the UK Carroll et al used GP records in London and found a prevalence of 8% of men and 5% of women over 44 years of age (19). There was a history of myocardial infarction in 30% of men and 22% of women. Lampe and colleagues examined trends in the prevalence of CHD in men participating in the BRHS (20). The authors demonstrated a decrease in the prevalence of current angina symptoms: the age adjusted annual percentage change in odds was -1.8%. However, there was no evidence of a trend in the prevalence of history of diagnosed CHD.

A study by Davies et al examined trends in CHD incidence, prevalence, and mortality in the UK between 1996 and 2005, using the THIN GP database (a total of 5 million patients). The results indicate that, while CHD mortality declined, CHD incidence decreased less than mortality, resulting in an increase in CHD prevalence (21). From 1996 to 2005, age-standardised incidence of CHD decreased by 2.2% in men and 2.3% in women per year (average percentage change). Age-standardised all-cause mortality among those with CHD decreased by 4.5% in men and 3.4% in women per year (average percentage change). Age-standardised prevalence increased by 1.3% in men and 1.7% in women per year (average percentage change). Although the decline in incidence had some impact on limiting the increase in prevalence, its effect was offset by the increase in prevalence occurring as a result of improved survival among people with CHD. Although patients with nitrate prescriptions were also included, this study relied mainly on CHD diagnostic codes which may underestimate actual prevalence.

2.1.3 Previous CHD prevalence modelling

An epidemiological CHD prevalence model was first developed in the UK to assist case-finding in Sheffield primary care trusts (PCTs). Subsequently, English PCTs were required to set targets for CHD case-finding in their 2007-8 Local Delivery Plans negotiated with strategic health authorities. To assist them a simple PCT-based prevalence model was developed rapidly by the Association of Public Health Observatories (APHO). Stage 1 of the modelling predicts the number of people with identified CHD within each population, taking account only of the demographic distribution of the population. The prevalence of patient-reported doctor-diagnosed CHD in each age/sex stratum is based on national data from the HSfE. Stage 2 takes account of deprivation levels in different PCTs in England. In the absence of sufficiently precise published data on the relationship between deprivation and CHD prevalence, the model makes the assumption that areas with higher CHD mortality rates have comparably higher prevalence of CHD. Using data for all local authorities in England, a linear relationship was calculated between 2002-04 SMRs for CHD and a deprivation score (UV67) derived from the 2001 Census Classification of Deprivation:

$$\text{CHD SMR} = (2.604 \times \text{UV67}) + 25.97$$

Using UV67 scores calculated for each PCT, the above formula gives a multiplying factor for each PCT. For example, a PCT with a UV67 score of 40% (very deprived) has a multiplying factor of 1.3.

APHO accepts that this model is rather crude, and that a prevalence model based on a comprehensive regression model using HSfE data would be more robust. This model was commissioned by APHO from the Department of Primary Care & Social Medicine at Imperial College London.

Congdon has also produced a CHD prevalence model using earlier HSfE data (22). Data from the 1999 and 2003 HSfEs were used to provide model-based rates of CHD prevalence by age, sex, ethnic group, region, and area deprivation category. To take into account the effect of socio-economic factors, the HSfE model gradient over deprivation quintiles was applied to scale area prevalence estimates specific for age, sex and ethnicity. The final stage of the prevalence estimation procedure incorporated proxy information from mortality. To adjust the HSfE-based prevalence rates to take account of interdependence of area mortality and prevalence, prevalence and mortality were taken as joint (i.e. correlated) outcomes in a Bayesian model allowing also for the spatial patterning of both outcomes, using WinBUGs software.

2.2 Methods

2.2.1 Data sources

The HSfE was used as the data source, and the outcome of interest was patient-reported doctor-diagnosed CHD (called IHD in HSfE), which is the variable used for CHD prevalence in HSfE. It was also chosen as on the basis of previous research it appears to be the best **single** proxy for true prevalence.

(An alternative would be to combine patient-reported doctor-diagnosed CHD with a positive Rose angina questionnaire as in BRHS.)

Because ethnicity is a known CHD risk factor, it was necessary to use a sample containing data from a large number of ethnic minority respondents. The HSfE 2004 was the last survey to include an ethnic minority boost, and the boost sample was used for the modelling. However there were relatively small numbers of whites in the HSfE 2004 sample, and they were not asked to respond to many questions, including those on CHD, presumably to save resources for the boost itself. The HSfE 2004 boost sample was therefore merged with the HSfE 2003, which was the year with the largest number of identical variables.

Table 3 shows the numbers of respondents in the original HSfE 2004 ethnic boost, and the number in the merged 2003-2004 dataset used for the model where a response for the CHD outcome variable was obtained. Note that it was necessary to collapse two of the HSfE 2003 ethnic group variables in order to use the same classification as HSfE 2004.

Table 3: Ethnic Group Breakdown of HSfE 2004 Dataset & Merged 2003-2004 Dataset

	HSfE 2004			Merged 2003-4 Dataset		
Ethnic Group	Freq.	Percent	Cum.	Freq.	Percent	Cum.
No answer/refused	21	0.21	0.21	16	0.08	0.08
Don't know	3	0.03	0.24	2	0.01	0.08
White	1,597	15.79	16.03	14,575	68.58	68.67
Mixed ethnic group	623	6.16	22.19	308	1.45	70.12
Black or Black British	2,468	24.40	46.59	1,991	9.37	79.48
Asian or Asian British	4,764	47.10	93.69	3,725	17.53	97.01
Any other group	638	6.31	100.00	635	2.99	100.00
Total	10,114	100.00		21,252	100.00	

(Note that there were 34 missing values.)

2.2.2 Model construction: data issues

The choice of variables for original inclusion in the merged dataset included all those known to be CHD risk factors. The variable names and labels are shown in Table 3 below. The HSfE dataset has a nested or hierarchical structure so three variables related to the sampling strata were included: area (sample point), cluster (stratification level), and hserial (serial number of household). These were used in the model to adjust for clustering of respondents. In the analysis variables cholest and hdlchol were combined to give a lipid ratio.

The bandings of Index of Multiple Deprivation 2004 scores were slightly different between the two years, but raw scores were not provided in the dataset so it was necessary to assume identity (see Table 4 below).

The Stata10 software package was used for analysis. All variables were re-coded to drop negative values for estimation purposes (in HSfE various non-response categories are assigned negative values). The methodology applied was multinomial logistic regression with the "cluster" adjustment option for households (see above). For analysis of two categories as here, multinomial logistic regression is reduced to binomial logistic regression. However the reason for not using other logistic regression routines that take into account nested structures is that the other options available in Stata produced an estimation error, probably because of the small percentage of disease-positive respondents in the sample.

Table 4: Index of Multiple Deprivation Banding

Rank	IMD Band	IMD		Number	Per Cent	Cum Per Cent
		HSfE 2003	HSfE 2004			
least	1	0.59-8.35	0.55-9.02	3,803	17.87	17.87
	2	8.35-13.72	9.03-14.14	3,573	16.79	34.65
	3	13.72-21.16	14.15-21.17	3,788	17.8	52.45
	4	21.16-34.21	21.18-33.52	4,551	21.38	73.83
most	5	34.21-86.36	33.53-85.69	5,571	26.17	100
			Total	21,286	100	

The modelling and estimation of the effects of interest was carried out using the mlogit command. The initial output consisted of two tables: one with the estimated regression coefficients, corresponding p-values and 95% confidence intervals, and another with the estimated odds ratios (exp(b)), which in the table appear as relative risk ratios (RRRs) and 95% confidence intervals. A positive sign of the estimated coefficient is associated with an increase in the odds of the outcome had angina or heart attack, and a negative sign is associated with a decrease in the odds. Since Prob (A) = Odds (A) / 1+ Odds (A), for uncommon outcomes such as CHD, RRR can be assumed to be the same as the odds ratio (OR).

For categorical variables the effects are estimated relative to the reference category. Stata uses the first category as reference (baseline OR). Separate baseline odds were estimated for each gender, and also according to ethnicity, age band, area-based deprivation score etc. The model was then used to derive the prevalence ratios for CHD for subjects with various combinations of risk factors in relation to baseline, using stepwise addition of variables. The prevalence in each age group, gender, ethnic group, area of residence and level of deprivation, and smoking status category were derived from the odds, using the formula: prevalence = odds/(1 + odds).

2.2.3 Model construction: interactions between variables

Effect modification or interaction occurs if the effect of one exposure or risk factor on the outcome varies according to the level of another risk factor. This can be tested using a χ^2 test of heterogeneity e.g. Mantel-Haenszel odds ratios, Wald or likelihood tests, or by introducing interaction terms or

parameters into the regression model. These allow the effect of one variable to be different in different categories of other variables. In Stata the xi command expands terms containing categorical variables into indicator (also called dummy) variable sets by creating new variables and estimates interactions and main effects.

An initial examination for interactions was carried out using Mantel-Haenszel odds ratios, Wald or likelihood ratio tests. Where these reached significance interaction terms were created as indicator variables in a regression model. None of the interaction terms tested in this way showed consistent statistical significance. The inclusion of interaction terms in the model was therefore rejected.

2.2.4 Model construction: internal validation

Ideally the best prediction should result from utilising the most risk factor information in the regression model. However only a limited range of HSfE variable data is either available or can be estimated at the primary care organisation (PCO) or local authority (LA) level, so there is no purpose in including other variables (see Table 5). We decided to validate the local model by comparing it, in terms of prediction, to a model including all available and significant HSfE variables. In addition, however, the amount of missing data affects the prediction of a model. In the complete HSfE variables the largest proportion of missing data occurred in those variables related to drug treatment for high blood pressure. We therefore excluded these variables in a second version of the model. Hence the model COMP 1 included the "complete" list of variables, including BP drugs; model COMP 2 included the "complete" list of variables, but excluded BP drugs.

LOCAL only used locally available data. We included smoking and BMI variables on the basis that local synthetic estimates are now available. Local GHQ-12 or Limiting Longstanding Illness score data will be available locally from 2009 and could be included later.

Table 5: Variables Included in Merged Dataset

COMP 1 = "Complete" variables with BP drugs
COMP 2 = "Complete" variables without BP drugs
LOCAL= only using locally available data

Name	Label	COMP 1	COMP 2	LOCAL
ihdis	had IHD (angina or heart attack)	X	X	X
aceinh	ace inhibitors (blood pressure)	X		
addnum	address number	N/A	N/A	N/A
adtot30	adults: total days/4 weeks active 30 mins + moderate +	X	X	
age	age last birthday	X	X	X
area	sample point	N/A	N/A	N/A
beta	beta blockers (blood pressure)	X		
bmival	valid body mass index	X	X	X
calciumb	calcium blockers (blood pressure)	X		
cholest	total cholesterol result (blood data)	X	X	
cigst1	cigarette smoking status - never/ex-reg/ex-occ/current	X	X	X

Name	Label	COMP 1	COMP 2	LOCAL
cluster	stratification level	N/A	N/A	N/A
diabete2	doctor diagnosed diabetes (excluding pregnant)	X	X	
diur	diuretics (blood pressure)	X		
ethnici	ethnic group	X	X	
famcvd	family history of cvd	X	X	
fatvala	fat score	X	X	
fldlchol	LDL cholesterol result (fasting blood data)	X	X	
ftrigl	triglycerides result (fasting)	X	X	
ghq12scr	General Health Questionnaire score- 12 point scale	X	X	
glucval	glucose result (fasting)	X	X	
hdlchol	HDL cholesterol result (blood data)	X	X	
hserial	serial number of household	N/A	N/A	N/A
imd2004	index of multiple deprivation (SOA level)	X	X	X
limitill	limiting longstanding illness	X	X	
nssec8	National Statistics Socioeconomic Class (8 variable)	X	X	
obpdrug	other drugs affecting BP	X		
omdiaval	omron valid mean diastolic BP	X	X	
omsysval	omron valid mean systolic BP	X	X	
porftvg	grouped portions of fruit (inc.orange juice) & veg yesterday	X	X	
roseanmi	angina or MI (Rose angina questionnaire)	N/A	N/A	N/A
sex	sex	X	X	X
topqual3	highest educational level	X	X	

The prediction of the three models was assessed in two ways:

- by generating a receiver operating characteristics (ROC) curve using the predicted probabilities of the CHD outcome compared to the observed cases
- by deriving predicted probabilities of the CHD outcome in Stata from the three models, and comparing these to the observed cases

2.2.5 Model construction: external validation

An early external validation will be carried out by examining the association between PCT/LA level CHD prevalence estimates and QOF registered prevalence. The regression-based model will also be validated against a prevalence model obtained by Bayesian strategies using WinBUGS. Finally, funding will be sought to undertake a validation of practice-based prevalence estimates against registered prevalence supplemented by active case finding.

2.3 The Model

Table 6 shows the frequency of the CHD outcome by age group in the merged dataset.

Table 6: Respondents Reporting Doctor Diagnosed CHD by Age Band

	Age Band							
	16-24	25-34	35-44	45-54	55-64	65-74	75+	Total
Don't know	0	0	0	0	0	1	2	3
Yes	2	6	43	97	262	350	344	1,104
No	2,500	3,855	4,409	3,379	2,792	1,928	1,316	20,179
Total	2,502	3,861	4,452	3,476	3,054	2,279	1,662	21,286

2.3.1 Local model

The regression model for risk factors for CHD in the "local" prevalence model is shown in Table 7. As expected ORs increase strikingly with increasing age in all models. In the prevalence predictions using coefficients (not shown in these tables) this results in age-related increases in prevalence which closely match the crude overall prevalences in Table 6. Surprisingly the only significant comparison for smoking is for category 3 "used to smoke regularly" i.e. this group is more likely to report CHD compared to the group "never smoked cigarettes at all". There is a significant comparison for male sex. ORs, p values and confidence intervals are generally similar to the "Complete" models. Unfortunately, however, local synthetic estimates of smoking prevalence do not include categories for occasional/regular smokers.

Table 7: Odds Ratios for LOCAL, Model With Only Locally Available Variables

Risk factor	RRR	Std Error	z	P>z	[95% Conf.	Interval]
Age 25-34	1.000					
Age 35-44	6.867	2.998	4.41	0	2.919	16.157
Age 45-54	19.514	8.217	7.06	0	8.549	44.541
Age 55-64	65.698	27.217	10.1	0	29.169	147.972
Age 65-74	122.864	50.719	11.65	0	54.707	275.936
Age 75+	191.252	79.128	12.7	0	85.003	430.307
Female sex	1.000					
Male sex	1.849	0.139	8.16	0	1.595	2.143
Never smoker	1.000					
Used to smoke occasionally	0.757	0.131	-1.61	0.107	0.539	1.062
Used to smoke regularly	1.484	0.119	4.91	0	1.267	1.737
Current smoker	1.072	0.109	0.68	0.495	0.878	1.308
Index of multiple deprivation 0.59-8.35	1.000					
Index of multiple deprivation 8.35-13.72	1.226	0.143	1.75	0.08	0.976	1.541
Index of multiple deprivation 13.73-21.16	1.350	0.154	2.63	0.009	1.079	1.689
Index of multiple deprivation 21.17-34.21	1.645	0.183	4.49	0	1.323	2.044
Index of multiple deprivation 34.22-86.36	2.420	0.256	8.36	0	1.967	2.978
White	1.000					
Mixed	1.264	0.851	0.35	0.727	0.338	4.726
Black/BB	0.763	0.168	-1.23	0.218	0.496	1.173

Asian/AO	1.511	0.243	2.56	0.01	1.102	2.071
Other	0.168	0.170	-1.76	0.079	0.023	1.227

We examined the data for interactions between variables e.g. if there is an interaction between sex and ethnicity, there will be two separate effects of ethnicity on CHD: one for males and another for females. We tested for interactions between local variables initially using Mantel-Haenszel odds ratios, and likelihood ratio and Wald tests. These indicated a possible interaction between sex and ethnicity. However a regression analysis showed significance for only one sex-ethnicity indicator level. We therefore decided not to include an interaction variable in the model.

2.3.2 Complete model excluding treatment for hypertension

The regression model for risk factors for CHD in the "Complete" model excluding hypertension (either systolic or diastolic BPs or treatment for hypertension) is shown in Table 9. This shows well the impact of additional variables on ORs for "local" variables. BMI is now recognised as an independent risk factor for CVD (although its effect is mediated largely through changes in "physiological" risk factors such as cholesterol:HDL ratio (23-25)). Although an expected higher OR is found in underweight patients, this is not the case for overweight/obese categories. This may be due to the fact that this is cross-sectional rather than longitudinal data and that the prevalence of obesity in older age groups, where CHD is more common, is very low.

Table 8: CHD prevalence by BMI category

	BMI <18.51	BMI >18.50 & BMI<25	BMI >25 & BMI <30	BMI >30 & BMI <40	BMI >40	Total
CHD	12	187	386	268	251	1,104
No CHD	306	6,693	6,502	3,483	3,194	20,178
Total	318	6,880	6,888	3,751	3,445	21,282

Table 9: Odds ratios for complete model excluding treatment for hypertension

Risk factor	RRR	Std Error	z	P>z	[95% Conf.	Interval]
Age 25-34	1.000					
Age 35-44	7.131	6.891	2.03	0.042	1.073	47.396
Age 45-54	18.447	17.436	3.08	0.002	2.893	117.624
Age 55-64	50.099	46.710	4.2	0	8.058	311.490
Age 65-74	121.219	112.515	5.17	0	19.655	747.578
Age 75+	216.341	200.711	5.8	0	35.111	1333.034
Female sex	1.000					
Male sex	2.258	0.320	5.74	0	1.710	2.981
Never smoker	1.000					

Risk factor	RRR	Std Error	z	P>z	[95% Conf.	Interval]
Used to smoke occasionally	1.039	0.320	0.13	0.9	0.569	1.900
Used to smoke regularly	1.512	0.238	2.63	0.009	1.111	2.057
Current smoker	1.114	0.232	0.52	0.604	0.740	1.676
Index of multiple deprivation 0.59->8.35	1.000					
Index of multiple deprivation 8.35-13.72	1.215	0.241	0.98	0.325	0.824	1.791
Index of multiple deprivation 13.73-21.16	1.025	0.217	0.12	0.907	0.677	1.551
Index of multiple deprivation 21.17-34.21	1.101	0.230	0.46	0.646	0.730	1.659
Index of multiple deprivation 34.22-86.36	1.424	0.323	1.56	0.119	0.913	2.219
White ethnic group	1.000					
Mixed ethnic group	0.208	0.144	-2.26	0.024	0.054	0.810
Black or Black British ethnic group	0.831	0.355	-0.43	0.666	0.360	1.922
Asian or Asian British ethnic group	1.553	0.436	1.57	0.117	0.896	2.693
Any other ethnic group	1.049	0.665	0.08	0.939	0.303	3.631
BMI <18.51	1.000					
BMI >18.50 & <25	0.538	0.568	-0.59	0.557	0.068	4.268
BMI >25 & BMI <30	0.890	0.936	-0.11	0.912	0.113	6.984
BMI >30 & BMI <40	1.026	1.085	0.02	0.98	0.129	8.143
BMI >40	0.693	0.734	-0.35	0.729	0.087	5.515
Total cholesterol:HDL ratio	0.748	0.049	-4.44	0	0.658	0.850
Diabetes; no	1.000					
Diabetes; yes	0.686	0.144	-1.8	0.072	0.455	1.035
Family History of CVD; yes	1.000					
Family History of CVD; no	0.622	0.103	-2.87	0.004	0.450	0.860
Limiting longstanding illness	1.000					
Non limiting longstanding illness	0.625	0.102	-2.89	0.004	0.454	0.859
No limiting longstanding illness	0.318	0.058	-6.24	0	0.222	0.456
Rose questionnaire: angina and MI	1.000					
Rose questionnaire: neither angina MI	0.007	0.004	-9.06	0	0.002	0.021
Rose questionnaire: angina, but not MI	0.074	0.044	-4.36	0	0.023	0.239
Rose questionnaire: MI, but not angina	0.174	0.096	-3.16	0.002	0.059	0.514
Top qualification nvq4/nvq5/degree	1.000					
Top qualification higher ed below degree	1.479	0.428	1.35	0.176	0.839	2.607
Top qualification nvq3/gce a level equiv	1.122	0.407	0.32	0.751	0.551	2.283
Top qualification nvq2/gce o level equiv	1.227	0.334	0.75	0.453	0.719	2.092
Top qualification nvq1/cse other grade equiv	1.443	0.504	1.05	0.294	0.728	2.861
Top qualification foreign/other	0.875	0.305	-0.38	0.701	0.442	1.732
Top qualification no qualification	1.458	0.355	1.55	0.121	0.905	2.349

2.3.3 Complete model including treatment for hypertension

The regression model for risk factors for CHD in the “complete” model with BP drugs is shown in Table 10. Hypertension is a well-established risk factor, so it is desirable to include variables related to it in a

"complete" model. Treatment with ACE inhibitors, beta blockers and calcium blockers are highly significant. However these may also be used to treat established CHD, so the association may be unrelated to hypertension. In addition, the HSfE relies upon patient recall for drug treatment. Not unexpectedly, much of the data for these variables is missing. We added systolic and diastolic BP as ordinal variables to the model, but this resulted in major changes to ORs for other variables. This may simply be related to model instability because of the large numbers of variables included. The addition of a single variable for hypertension, either treated or untreated, will be explored as a further later step in model development.

Table 10: Odds ratios for complete model including treatment for hypertension

Risk factor	RRR	Std Error	z	P>z	[95% Conf.	Interval]
Age 25-34	1.000					
Age 35-44	3.975	3.522	1.56	0.119	0.700	22.566
Age 45-54	8.606	7.259	2.55	0.011	1.647	44.959
Age 55-64	18.559	15.529	3.49	0	3.600	95.676
Age 65-74	36.219	30.104	4.32	0	7.103	184.684
Age 75+	61.082	50.587	4.97	0	12.050	309.643
Female sex	1.000					
Male sex	2.273	0.360	5.18	0	1.666	3.101
Never smoker	1.000					
Used to smoke occasionally	0.989	0.342	-0.03	0.975	0.502	1.948
Used to smoke regularly	1.450	0.246	2.18	0.029	1.039	2.023
Current smoker	1.201	0.278	0.79	0.43	0.762	1.892
Index of multiple deprivation 0.59->8.35	1.000					
Index of multiple deprivation 8.35-13.72	1.214	0.273	0.86	0.39	0.781	1.887
Index of multiple deprivation 13.73-21.16	1.033	0.248	0.13	0.893	0.645	1.653
Index of multiple deprivation 21.17-34.21	1.174	0.270	0.7	0.485	0.748	1.843
Index of multiple deprivation 34.22-86.36	1.394	0.366	1.26	0.206	0.833	2.331
White ethnic group						
Mixed ethnic group	0.353	0.240	-1.53	0.125	0.093	1.336
Black or Black British ethnic group	0.304	0.169	-2.14	0.032	0.102	0.902
Asian or Asian British ethnic group	1.316	0.391	0.92	0.356	0.734	2.357
Any other ethnic group	0.894	0.816	-0.12	0.902	0.149	5.345
BMI <18.51	1.000					
BMI >18.50 & <25	0.435	0.441	-0.82	0.411	0.060	3.171
BMI >25 & BMI <30	0.588	0.591	-0.53	0.597	0.082	4.218
BMI >30 & BMI <40	0.680	0.689	-0.38	0.704	0.093	4.954
BMI >40	0.519	0.525	-0.65	0.517	0.072	3.763
Total cholesterol:HDL ratio	0.742	0.053	-4.14	0	0.644	0.854
Diabetes; no	1.000					
Diabetes; yes	1.034	0.250	0.14	0.888	0.645	1.660
Family History of CVD; yes	1.000					

Risk factor	RRR	Std Error	z	P>z	[95% Conf.	Interval]
Family History of CVD; no	0.630	0.115	-2.54	0.011	0.441	0.900
Limiting longstanding illness	1.000					
Non limiting longstanding illness	0.592	0.102	-3.05	0.002	0.422	0.829
No limiting longstanding illness	0.578	0.128	-2.48	0.013	0.375	0.892
Rose questionnaire: angina and MI	1.000					
Rose questionnaire: neither angina MI	0.007	0.004	-8.49	0	0.002	0.022
Rose questionnaire: angina, but not MI	0.063	0.039	-4.4	0	0.018	0.215
Rose questionnaire: MI, but not angina	0.182	0.107	-2.91	0.004	0.058	0.574
Top qualification nvq4/nvq5/degree	1.000					
Top qualification higher ed below degree	1.655	0.549	1.52	0.129	0.864	3.173
Top qualification nvq3/gce a level equiv	1.396	0.593	0.79	0.432	0.607	3.211
Top qualification nvq2/gce o level equiv	1.510	0.457	1.36	0.173	0.834	2.733
Top qualification nvq1/cse other grade equiv	1.768	0.665	1.52	0.129	0.847	3.694
Top qualification foreign/other	0.922	0.371	-0.2	0.84	0.419	2.031
Top qualification no qualification	1.496	0.408	1.48	0.14	0.876	2.553
ACE inhibitor: no	1.000					
ACE inhibitor: yes	1.875	0.335	3.52	0	1.321	2.661
Beta blocker: no	1.000					
Beta blocker: yes	3.467	0.570	7.57	0	2.512	4.785
Calcium blocker: no	1.000					
Calcium blocker: yes	2.735	0.468	5.88	0	1.956	3.825
Diuretic: no	1.000					
Diuretic: yes	0.832	0.140	-1.09	0.276	0.598	1.158
Other BP drug: no	1.000					
Other BP drug: yes	0.869	0.279	-0.44	0.662	0.463	1.631

2.3.4 Validation: area under Receiver Operating Characteristics curve (AUROC)

Receiver-operating characteristic (ROC) analysis was originally developed during World War II to analyse classification accuracy in differentiating signal from noise in radar detection. Recently, the methodology has been adapted to several clinical areas heavily dependent on screening and diagnostic tests, in particular, laboratory testing, epidemiology, radiology, and bioinformatics (26;27). ROC analysis is a useful tool for evaluating the performance of diagnostic tests and more generally for evaluating the accuracy of a statistical model (e.g. logistic regression, linear discriminant analysis) that classifies subjects into one of two categories, diseased or non-diseased, as in this model (28). Its function as a simple graphical tool for displaying the accuracy of a medical diagnostic test is one of the most well-known applications of ROC curve analysis.

An ROC curve is a plot of sensitivity on the *y* axis against (1-specificity) on the *x* axis for varying values of the threshold, t. The 45° diagonal line connecting (0,0) to (1,1) is the ROC curve corresponding to

random chance. The ROC curve for the gold standard is the line connecting (0,0) to (0,1) and (0,1) to (1,1). Generally, ROC curves lie between these two extremes. The area under the ROC curve is a summary measure that essentially averages diagnostic accuracy across the spectrum of test values. The area under the curve (AUC) is an overall summary of diagnostic accuracy. AUC equals 0.5 when the ROC curve corresponds to random chance and 1.0 for perfect accuracy. On rare occasions, the estimated AUC is <0.5, indicating that the test does worse than chance.

AUROCs for the three models tested above were estimated using Stata10. These are shown in the chart below. If both sensitivity and specificity are of importance in a CHD model, the optimal threshold of t would be 0.75, where sensitivity and specificity equal 0.77. The local model exceeds this level, although the two more complete models have even better performance.

Comparison of AUROC for different CHD prevalence models

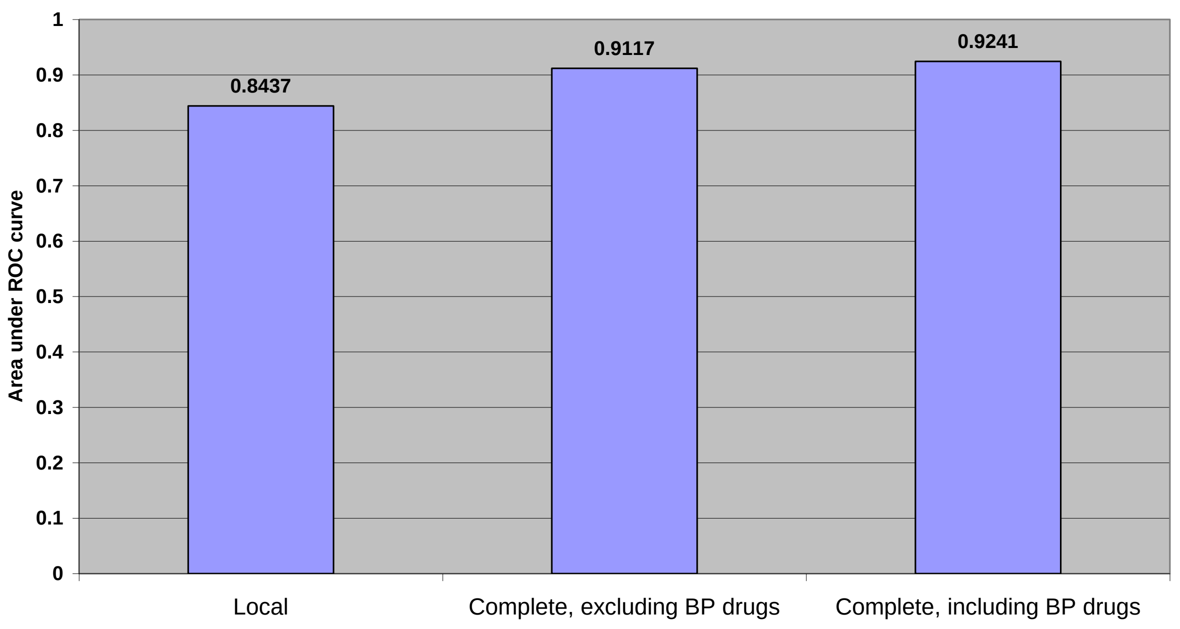

2.3.5 Validation: model prediction

Another method of assessing performance is to use the regression model to predict the response for each subject. These predictions are called fitted values. The difference between the fitted and the observed values are called residuals. These can then be tabulated against the observed presence of CHD to assess “misclassification” by each model. The results for the three models are shown in table 11.

Table 11: Comparison of predictions by model

	Observed	Predicted		
Model		No CHD	CHD	Total

Local	No CHD	17,614	3	17,617
	CHD	1,098	1	1,099
	Total	18,712	4	18,716
Complete, no BP drugs	No CHD	3,879	64	3,943
	CHD	1	230	219
	Total	4,109	283	4,392
Complete	No CHD	3,883	60	3,943
	CHD	203	246	449
	Total	4,086	306	4,392

3 Application of the Model

The local model (which includes only those variables that are available at population level i.e. age, sex, ethnicity, smoking status and deprivation score) has been applied to Local Authorities to create prevalence estimates of hypertension in those aged 16+ for 2005–2020. Models for PCTs have been created for 2006-2020.

3.1 Assumptions of the modelled estimates

It is assumed that:

- the proportion of smokers, ex-smokers and never-smokers is the same across ethnic groups.
- the proportion of ex-smokers in each age-sex group is the same in all areas.
- the smoking prevalence rates from the model-based estimates of lifestyle behaviours (27) are reliable.
- the prevalence of CHD in those aged 16-24 is negligible.

Due to lack of data, it was not possible to treat ex-regular-smokers and ex-occasional-smokers separately. Ex-occasional-smokers are treated as non-smokers.

3.2 Input data

3.2.1 Populations

3.2.1.1 Local Authorities

The CHD prevalence model uses ONS 2005 mid-year population estimates by ethnic group, age and sex. ONS publishes the data by broad age band (28), but supplied full quinary age-band data to APHO for the prevalence modelling project. Five ethnic groups were used: white, black, Asian, mixed and other.

3.2.1.2 Primary Care Trusts

The CHD prevalence model uses ONS 2006 mid-year population estimates by ethnic group, age and sex. ONS publishes the data by broad age band (28), but supplied full quinary age-band data to APHO for the prevalence modelling project. Five ethnic groups were used: white, black, Asian, mixed and other.

3.2.1.3 Population projections

In order to calculated estimate prevalence of CHD in the future, population projections were incorporated into the model. ONS has not published population projections by ethnic group, so the 2005 (LA) or 2006 (PCT) distribution of ethnic groups was used to generate population estimates to 2020.

For 2006 and 2007 LA population projections the distribution of ethnic groups in 2005 was applied to ONS mid-year population estimates by quinary age band. For 2007 PCT population projections the distribution of ethnic groups in 2006 was applied to ONS mid-year population estimates by quinary age band.

For 2008 and 2009 the distribution of ethnic groups in 2005 (LA) or 2006 (PCT) was applied to ONS 2006-based population projections by quinary age band for LAs and PCTs.

For 2010, 2015 and 2020 the distribution of ethnic groups in 2005 (LA) or 2006 (PCT) was lagged by 5, 10 and 15 years respectively and combined with ONS 2006-based population projections by quinary age band for LAs and PCTs. For example, the ethnic proportions for the age band 40-45 in 2005 were applied to the population aged 50-55 in 2015. With the exception of this 'ageing' of the ethnic population, no other changes to the distribution of ethnic groups were considered.

3.2.2 Smoking status

National (England) proportions of smokers, ex-smokers and current smokers by age and sex are taken from HSfE (2003-2005 pooled). These proportions were then adjusted for each LA/PCT using the synthetic estimates of smoking prevalence for 2003-2005 (27), using the following algorithm.

Local proportion of smokers in age-sex category = national prevalence of smoking in age-sex category * local overall smoking prevalence / national overall smoking prevalence

$$S_{asl} = S_{asn} \times \frac{S_l}{S_n}$$

Local proportion of ex-smokers in age-sex category is not adjusted

$$E_{asl} = E_{asn}$$

Local proportion of never-smokers in age-sex category = 1 – proportion of ex-smokers in age-sex category – local proportion of smokers in age-sex category

$$N_{asl} = 1 - E_{asl} - S_{asl}$$

Where:

S = proportion of population who are smokers
E = proportion of population who are ex-regular-smokers
N = proportion of population who have never smoked

l = local
n = national
as = by age and sex

This approach assumes that the proportion of ex-smokers in each age-sex category is fixed and the number of never-smokers increases as the number of smokers decreases. Regional analysis of the relationship between prevalence of smokers and ex-smokers in the Health Survey for England shows no systematic relationship and therefore it was decided that the ex-smoking rate should not be locally adjusted.

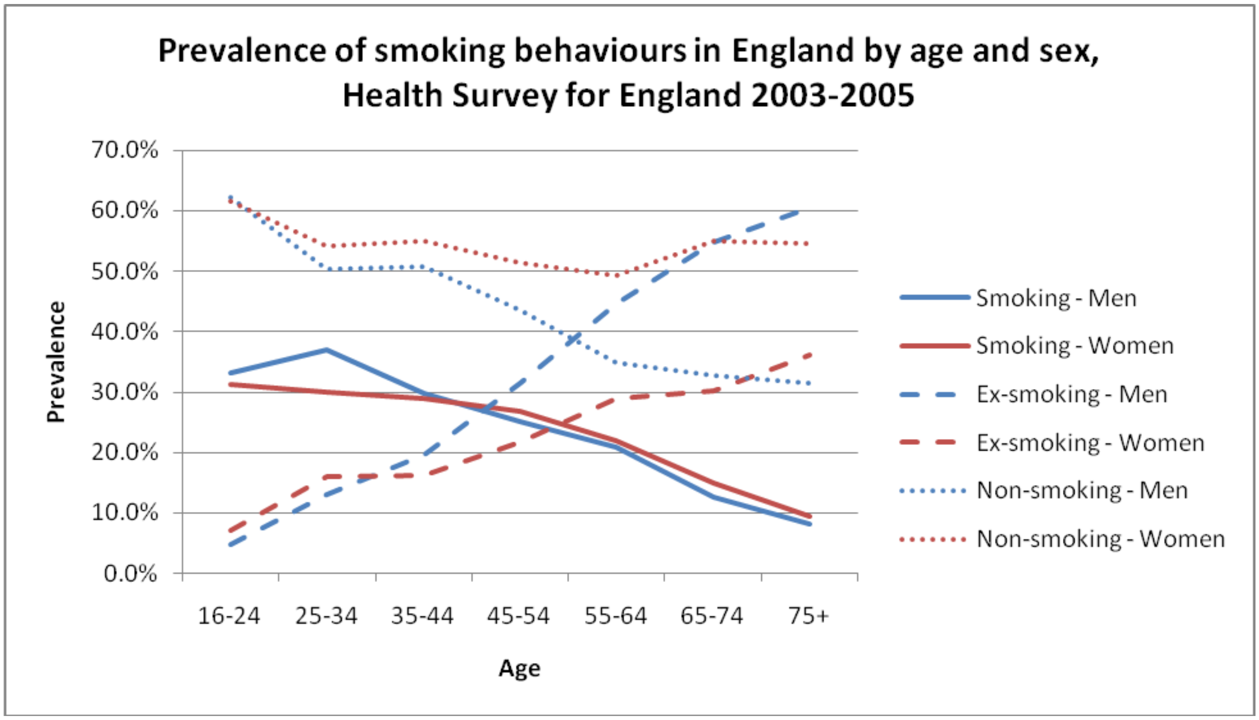

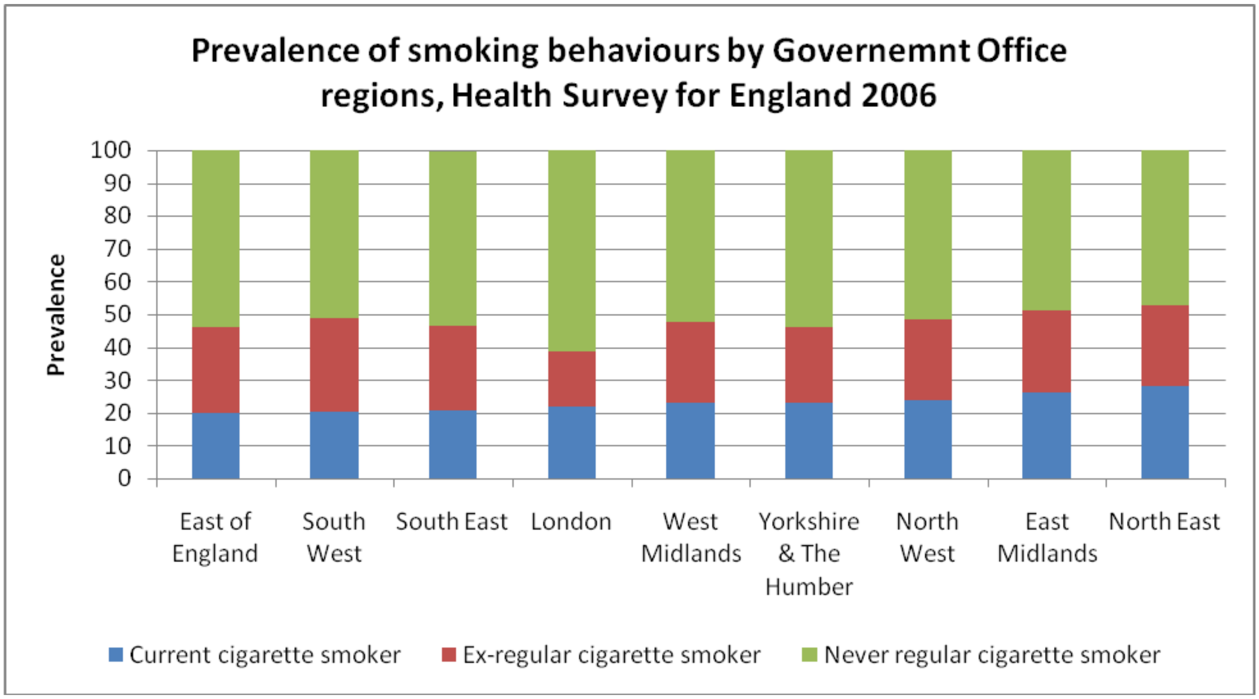

The same smoking prevalence rates are applied across all ethnic categories.

Future changes in smoking prevalence are not taken into account in the CHD prevalence projections. This is because of the uncertainty associated with predictions of smoking prevalence, and the lag time between smoking cessation and improved health. Even if there was a rapid drop in the number of smokers over the next few years, any associated decrease in CHD would not be seen for many years.

3.2.3 Deprivation

Deprivation scores are taken from IMD 2004 (29). Deprivation scores for PCTs were calculated by taking a population weighted average of the scores for each MSOA (which in turn were calculated by taking a weighted average of the IMD2004 scores of each LSOA) within the PCT.

Five deprivation categories are used in the model. Note that these categories are based on quintiles of IMD score at LSOA level. When the cut-offs are applied to larger geographies (LA or PCT) there is not an even distribution across the categories.

4 References

(1) Disease prevalence models to support 2007-8 PCT Local Delivery Plans (LDPs), APHO

(2) The Information Centre. Health Survey for England 2006 Latest Trends, Table 12: Prevalence of IHD, stroke, IHD or stroke (ever), by survey year, age and sex. The Information Centre 2008;Available from: URL: http://www.ic.nhs.uk/webfiles/publications/HSE06/ADULT%20TREND%20TABLES%202006.xls

(3) The Information Centre. Quality and Outcomes Framework (QOF) for April 2006 to March 2007, England: Numbers of patients on QOF disease registers, and unadjusted prevalence rates. The Information Centre 2008;Available from: URL: http://www.ic.nhs.uk/webfiles/QOF/2006-07/National%20QOF%20tables%202006-07%20-%20prevalence.xls

(4) De Bacquer D, De Backer G, Kornitzer M. Prevalences of ECG findings in large population based samples of men and women. Heart 2000 December 1;84(6):625-33.

(5) Lampe FC, Walker M, Lennon LT, Whincup PH, Ebrahim S. Validity of a Self-reported History of Doctor-diagnosed Angina. Journal of Clinical Epidemiology 1999 January;52(1):73-81.

(6) Walker M, Shaper AG, Wannamethee SG, Whincup PH. Measuring the prevalence of disease in middle-aged British men. Journal of the Royal College of Physicians of London 1999 July 1;33(4):351-8.

(7) Shaper AG, Cook DG, Walker M, Macfarlane PW. Prevalence of ischaemic heart disease in middle aged British men. Br Heart J 1984 June 1;51(6):595-605.

(8) Shaper AG, Cook DG, Walker M, Macfarlane PW. Recall of diagnosis by men with ischaemic heart disease. Br Heart J 1984 June 1;51(6):606-11.

(9) Lampe FC, Morris RW, Whincup PH, Walker M, Ebrahim S, Shaper AG. Is the prevalence of coronary heart disease falling in British men? Heart 2001 November 1;86(5):499-505.

(10) Information Centre for Health & Social Care. Health Survey for England 2006 Latest Trends. Information Centre for Health & Social Care 2008 January 31;Available from: URL: http://www.ic.nhs.uk/statistics-and-data-collections/healthand-lifestyles-related-surveys/health-survey-for-england/health-survey-for-england-2006-latest-trends

(11) Alexander CM, Landsman PB, Teutsch SM, Haffner SM. NCEP-defined metabolic syndrome, diabetes, and prevalence of coronary heart disease among NHANES III participants age 50 years and older. Diabetes 2003;52(5):1210.

(12) Ford ES, Giles WH, Croft JB. Prevalence of nonfatal coronary heart disease among American adults. The American heart journal 2000;139(3):371-7.

(13) Goldman L, Phillips KA, Coxson P, Goldman PA, Williams L, Hunink MGM, Weinstein MC. The effect of risk factor reductions between 1981 and 1990 on coronary heart disease incidence, prevalence, mortality and cost. Journal of the American College of Cardiology 2001 October;38(4):1012-7.

(14) Tunstall-Pedoe H. Contribution of trends in survival and coronary-event rates to changes in coronary heart disease mortality: 10-year results from 37 WHO MONICA project populations. Monitoring trends and determinants in cardiovascular disease. Lancet, The 1999;353(9164):1547-57.

(15) Gupta R, Prakash H, Gupta VP, Gupta KD. Prevalence and determinants of coronary heart disease in a rural population of India. Journal of Clinical Epidemiology 1997 February;50(2):203-9.

(16) AHTO MERJ, ISOAHO RAIM, PUOLIJOKI HANN, LAIPPALA PEKK, ROMO MATT, KIVELA SL. Prevalence of coronary heart disease, associated manifestations and electrocardiographic findings in elderly Finns. Age Ageing 1998 November 1;27(6):729-38.

(17) Hemingway H, Langenberg C, Damant J, Frost C, Pyorala K, Barrett-Connor E. Prevalence of Angina in Women Versus Men: A Systematic Review and Meta-Analysis of International Variations Across 31 Countries. Circulation 2008 March 25;117(12):1526-36.

(18) Carroll K, Majeed A, Firth C, Gray J. Prevalence and management of coronary heart disease in primary care: population-based cross-sectional study using a disease register. J Public Health 2003 March 1;25(1):29-35.

(19) Davies AR, Smeeth L, Grundy EMD. Contribution of changes in incidence and mortality to trends in the prevalence of coronary heart disease in the UK: 1996 2005. Eur Heart J 2007 September 1;28(17):2142-7.

(20) Congdon P. Estimating CHD prevalence by small area: integrating information from health surveys and area mortality. Health & place 2008;14(1):59-75.

(21) Hippisley-Cox J, Coupland C, Vinogradova Y, Robson J, May M, Brindle P. Derivation and validation of QRISK, a new cardiovascular disease risk score for the United Kingdom: prospective open cohort study. BMJ 2007 July 21;335(7611):136.

(22) Hippisley-Cox J, Coupland C, Vinogradova Y, Robson J, Minhas R, Sheikh A, Brindle P. Predicting cardiovascular risk in England and Wales: prospective derivation and validation of QRISK2. BMJ 2008 June 23;336(7659):1475-82.

(23) Mozaffarian D, Wilson PWF, Kannel WB. Beyond Established and Novel Risk Factors: Lifestyle Risk Factors for Cardiovascular Disease. Circulation 2008 June 10;117(23):3031-8.

(24) Hanley JA, McNeil BJ. The meaning and use of the area under a receiver operating characteristic (ROC) curve. Radiology 1982 April 1;143(1):29-36.

(25) Hanley JA, McNeil BJ. A method of comparing the areas under receiver operating characteristic curves derived from the same cases. Radiology 1983 September 1;148(3):839-43.

(26) Zou KH, O'Malley AJ, Mauri L. Receiver-Operating Characteristic Analysis for Evaluating Diagnostic Tests and Predictive Models. Circulation 2007 February 6;115(5):654-7.

(27) Model based estimates of healthy lifestyle behaviours http://www.ic.nhs.uk/statistics-and-data-collections/population-and-geography/neighbourhood-statistics/neighbourhood-statistics:-model-based-estimates-of-healthy-lifestyle-behaviours-at-pco-level-2003-05

(28) ONS population estimates by ethnic group http://www.statistics.gov.uk/STATBASE/Product.asp?vlnk=14238

(29) IMD 2004 http://www.communities.gov.uk/archived/general-content/communities/indicesofdeprivation/216309/
