# Supplementary material for: Variations in cardiovascular disease under-diagnosis in England: national cross-sectional spatial analysis
Source: BMC Cardiovasc Disord. 2011 Mar 17;11:12. doi: 10.1186/1471-2261-11-12 (PMC3070686; doi:10.1186/1471-2261-11-12)
Supplement: Additional file 2 — Hypertension prevalence modelling briefing document v2. This document describes how the hypertension prevalence model was developed from Health Survey for England data and how the model was applied to local population data. [file 1471-2261-11-12-S2.PDF]

Hypertension Prevalence Modelling Briefing Document

Hannah Walford, ERPHO

Michael Soljak and Ruthie Birger, Department of Primary Care and Social Medicine & Statistical Advisory Service, Imperial College London

November 2008

This briefing document explains how the hypertension prevalence model has been developed and applied. It accompanies the hypertension prevalence estimates released in September 2008.

Contents

1 Background ... 3
2 Model development ... 3
2.1 Background ... 3
2.1.1 Defining Prevalence ... 5
2.2 Methods ... 6
2.2.1 Data Sources ... 6
2.2.2 Model construction: data issues ... 8
2.2.3 Model construction: interactions between variables ... 10
2.2.4 Model construction: internal validation ... 10
2.2.5 Model construction: external validation ... 11
2.3 The model ... 11
2.3.1 Local model ... 11
2.3.2 Complete model ... 13
2.4 Validation: area under Receiver Operating Characteristics curve (AUROC) ... 16
2.4.1 Validation: model prediction ... 18
3 Application of the Model ... 18
3.1 Assumptions of the modelled estimates ... 18
3.2 Input data ... 19
3.2.1 Populations ... 19
3.2.2 Deprivation ... 19
4 References ... 20

1 Background

A crude epidemiologic hypertension model based on age and sex-specific prevalence was first developed in the UK by the Faculty of Public Health (1). Subsequently, English PCTs were required to set targets for hypertension case-finding in their 2007-8 Local Delivery Plans negotiated with strategic health authorities. To assist them a simple PCT-based prevalence model was developed rapidly by the Association of Public Health Observatories (APHO).

In the APHO model numbers of persons predicted to be hypertensive were derived by multiplying April 2006 PCT registered populations by hypertension prevalence rates identified in the 2003 and 2004 HSfE, modified by ethnic-group age-standardised risk ratios from the 2004 HSfE. Calculations were stratified to reflect variations in these factors at PCT level. The PCT-registered populations were derived by aggregating April 2006 GP practice populations from the Exeter System using a practice to new PCT lookup. In the absence of age by sex by ethnic-group PCT populations, the age by sex registered populations of the current PCTs were attributed the ethnic-group distributions of their constituent former PCT/s resident populations from the 2001 Census.

APHO accepts that the model was rather crude, and that a prevalence model based on a comprehensive regression model using HSfE data would be more robust. This new model was commissioned from the Department of Primary Care & Social Medicine at Imperial College London.

2 Model development

2.1 Background

Hypertension is defined as a persistently raised blood pressure (BP) above a designated threshold (which has reduced over time). The current NICE guidance (2) defines this further as follows:

- measured at the past two GP visits i.e. an average and
- systolic or diastolic pressure (SBP/DBP) or both are above 140/90 mmHg.

Hypertension is an important public health challenge in both economically developing, and developed, countries. Worldwide it is estimated that hypertension caused 7.6 million premature deaths in 2001 (3). Hypertension is one of the main risk factors for heart attack and stroke (4). Numerous national and regional studies have been conducted worldwide to estimate the prevalence of hypertension: hypertension rates have varied from 3.4% in rural Indian men to as high as 72.5% in Polish women (5).

Within developed countries, it appears that average BP is higher in Europe than North America, even adjusting for treatment differences (6). A meta-analysis of national studies showed a mean BP of 136/83 mm Hg in six European countries and 127/77 mm Hg in Canada and the United States among men and women combined who were 35 to 74 years of age. This difference already existed among

younger persons (35-39 years) in whom treatment was uncommon (i.e. 124/78 mm Hg and 115/75 mm Hg, respectively), and the slope with age was steeper in the European countries. For all age groups, BP measurements were lowest in the United States and highest in Germany. The age- and sex-adjusted prevalence of hypertension was 28% in the North American countries and 44% in the European countries at the 140/90 mm Hg threshold.

Hypertension is more common among African Caribbeans and South Asians in Britain. The table below from HSfE 2004 report shows that Black Africans and Caribbeans have the highest standardised risk ratios for hypertension. A number of studies in the UK have also shown that mortality rates for stroke, coronary heart disease (high blood pressure) and end-stage renal failure are higher in South Asians than white Europeans (5). The NICE guidelines recommend a reduction of BP to <140/85, but give lower target BP levels (<130/80) for patients with diabetes or chronic renal failure. The majority of UK-based surveys have shown that people of Black African or Black Caribbean origin have higher average BP levels and rates of hypertension than their white counterparts.

Table 7.8

Blood pressure levels, by minority ethnic group and sex

Aged 16 and over with valid blood pressure readings — *2004*

Blood pressure levels	Minority ethnic group							General population (2003)[a]
	Black Caribbean	Black African	Indian	Pakistani	Bangladeshi	Chinese	Irish	
**Men**								
**Observed %**								
Normotensive untreated[b]	62	75	67	80	84	80	64	68
Hypertensive controlled[b]	10	4	11	6	3	4	7	5
Hypertensive uncontrolled[b]	10	5	6	2	6	5	7	6
Hypertensive untreated[b]	19	16	16	11	7	11	23	20
All with hypertension	*38*	*25*	*33*	*20*	*16*	*20*	*36*	*32*
Hypertensive untreated[c]	2	4	3	2	1	2	5	5
**Standardised risk ratios**								
All with hypertension	1.37	1.21	1.15	0.98	0.63	0.78	1.13	1
Standard error of the ratio	0.19	0.22	0.13	0.19	0.14	0.13	0.18	
**Women**								
**Observed %**								
Normotensive untreated[b]	68	81	82	85	81	84	71	71
Hypertensive controlled[b]	9	5	4	4	6	6	4	6
Hypertensive uncontrolled[b]	10	2	6	5	5	3	9	8
Hypertensive untreated[b]	13	12	7	5	7	7	15	16
All with hypertension	*32*	*19*	*18*	*15*	*19*	*16*	*29*	*29*
Hypertensive untreated[c]	1	4	3	3	3	2	3	5
**Standardised risk ratios**								
All with hypertension	1.58	1.71	0.91	1.01	1.43	1.12	0.95	1
Standard error of the ratio	0.19	0.37	0.12	0.18	0.22	0.20	0.14	
Bases (weighted)								
Men	*169*	*136*	*361*	*159*	*53*	*63*	*667*	*4420*
Women	*249*	*183*	*442*	*207*	*82*	*66*	*923*	*4702*
Bases (unweighted)								
Men	*155*	*123*	*265*	*162*	*99*	*153*	*240*	*4108*
Women	*243*	*154*	*320*	*207*	*144*	*166*	*328*	*5075*

Source: HSfE 2004

Data from randomized controlled trials, in which mean age at event was approximately 70 years, indicate that a 10 mm Hg reduction in systolic BP is associated with a reduction in risk of stroke of approximately one third (4). Epidemiological, clinical and animal experimental evidence show a direct relationship between dietary electrolyte consumption and blood pressure. Clinical trials have shown that reduction of salt (sodium chloride, NaCl) intake reduces BP levels in normotensive and hypertensive populations and prevents the development of hypertension. Drug treatment is recommended in all people with sustained levels of blood pressure ≥160/100, and at lower levels in those with additional risk factors, such as diabetes, or with end-organ damage. The threshold for offering drug treatment is defined by NICE (2) as:

- blood pressure of more than 160/100 mmHg or
- isolated systolic hypertension (systolic blood pressure of more than 160 mmHg) or
- blood pressure of more than 140/90 mmHg and:
  - 10-year CVD risk of at least 20%, or
  - existing CVD or target organ damage.

2.1.1 Defining Prevalence

The prevalence of various categories of hypertension is shown in Table 1 below, taken from the HSfE 2003.

Table 1: Prevalence of Hypertensive Categories in HSfE 2003

(Adults aged 16 and over with a valid blood pressure reading and data on medication)

	16-24	25-34	35-44	45-54	55-64	65-74	75+	Total
**2003 (unweighted)[c]**								
Normotensive untreated	94.3	91.4	84.9	69.8	50.2	33.2	26.8	65.6
Normotensive treated	0.1	0.2	1.5	5.3	10.2	14.3	10.7	5.9
Hypertensive treated	-	0.3	1.0	3.5	11.5	19.4	26.1	8.0
Hypertensive untreated	5.5	8.0	12.6	21.4	28.1	33.0	36.4	20.5
All with high blood pressure	5.7	8.6	15.1	30.2	49.8	66.8	73.2	34.4
**2003 (weighted)[b]**								
Normotensive untreated	93.9	90.7	84.1	69.6	49.3	33.1	27.1	67.9
Normotensive treated	0.1	0.2	1.6	5.3	10.5	14.9	10.4	5.4
Hypertensive treated	-	0.4	1.0	3.4	11.9	19.5	26.9	7.3
Hypertensive untreated	6.0	8.8	13.3	21.7	28.3	32.6	35.5	19.4
All with high blood pressure	6.1	9.3	15.9	30.4	50.7	66.9	72.9	32.1
Bases								
2003 (unweighted)	849	1272	1800	1536	1625	1194	907	9183

	16-24	25-34	35-44	45-54	55-64	65-74	75+	Total
2003 (weighted)	1201	1460	1765	1457	1346	1032	861	9122

2.2 Methods

2.2.1 Data Sources

The hypertension model described here uses data from the 2003 and 2004 Health Surveys for England (HSfE). The 2003 Survey contains data on a total of 18,553 individuals. The 2004 data consists of two individual level files of which one (10,114 records) contains data for all individuals in the Ethnic Boost Sample and informants in the General Population Sample who were of the specified ethnic groups in co-operating households who gave a full interview. It contains information from the household questionnaire, main individual schedule, self-completions and the nurse visit (where one occurred). Data on under 16s was dropped from both these raw data files for the regression modelling.

Because hypertension prevalence is known to vary with ethnicity, it was necessary to use a sample containing data from a large number of ethnic minority respondents. The HSfE 2004 was the last Survey to include an ethnic minority boost, and the boost sample was used for the modelling. However there were relatively small numbers of Whites in the HSfE 2004 sample, and BP was not measured for most respondents in the 2004 general population sample, presumably to save resources for the boost itself. The HSfE 2004 boost sample was therefore merged with the HSfE 2003 data, which measured BP and which was the year with the largest number of identical variables. Table 2 shows the ethnic group breakdown of the two samples. Note that it was necessary to collapse two of the HSfE 2003 ethnic group variables in order to use the same classification as HSfE 2004.

Table 2: Ethnic Group Breakdown of HSfE 2003 & 2004 Datasets & Merged 2003-2004 Dataset

	HSfE 2003		HSfE 2004		HSfE 2003+2004	
	**Freq.**	**Percent**	**Freq.**	**Percent**	**Freq.**	**Percent**
white	13,445	92.11	1,130	16.98	14,575	68.64
mixed ethnic group	86	0.59	222	3.34	308	1.45
black or black british	308	2.11	1,683	25.29	1,991	9.38
asian or asian british	594	4.07	3,131	47.04	3,725	17.54
any other group	163	1.12	472	7.09	635	2.99
**Total**	**14,596**	**100**	**6,638**	**100**	**21,234**	**100**

NB excludes no answer/refused/don't know

In HSfE 2003, a new oscillometric automated device, the Omron HEM 907, was introduced to measure blood pressure, as a replacement for the Dinamap 8100, which had become obsolete. It should be noted that HSE 2004 measures blood pressure as a one-off measurement in a cross sectional survey and does not provide information on whether high BP is sustained over time (the clinical definition in the NICE

guidance), so this survey may overestimate the prevalence of hypertension. On the other hand, BP measurement by GPs or practice nurses may increase BP (so-called “white coat hypertension”). The HSfE 2004 report introduced a change to a clearer terminology from previous years’ reports. However, the actual definition did not change. The specific definitions of the four levels used in the HSE 2004 report are:

Normotensive untreated SBP <140mmHg and DBP <90mmHg and not taking medicine prescribed for high blood pressure

Hypertensive controlled SBP <140mmHg and DBP <90mmHg and taking medicine prescribed for high blood pressure (in 2003 this was called “normotensive-treated”)

Hypertensive uncontrolled SBP ≥140mmHg and/or DBP ≥90mmHg and taking medicine prescribed for high blood pressure (in 2003 this was called “hypertensive-treated”)

Hypertensive untreated SBP ≥140mmHg and/or DBP ≥90mmHg and not taking medicine prescribed for high blood pressure (in 2003 this was called “hypertensive-untreated”)

In HSfE 2003, valid blood pressure readings were obtained from 80.8% of men and 80.3% of women aged 16 and over who were visited by a nurse. Response rates increased with age for both sexes. The main reason for exclusion was that the informant ate, drank or smoked in the half hour prior to the measurement (17.0% among men and 15.2% among women). The remainder were excluded because they were pregnant, (4.9% of women aged 16-44 were excluded due to pregnancy) or because three valid readings had not been obtained or the measurement had been refused or not attempted.

In 2004, nurse visits were offered to all individuals in minority ethnic groups in the boost sample (note that the general population data presented in the HSfE 2004 report are actually from HSE 2003, as the general population did not receive a nurse visit in 2004). Response rates for BP measurements and urine samples are based on those receiving a nurse visit, though women who were pregnant were not asked for a urine sample. Section 6.3 in Volume 2 of the 2004 report gives more details response rates to the different stages of the survey. Among those who participated in a nurse visit, the proportion with three valid blood pressure readings ranged from 69% of Bangladeshi men, to 84% of Chinese and Indian men. Among women, the proportion with three valid blood pressure readings ranged from 71% of Black African women to 84% of Indian women. As in 2003, informants were excluded if they were pregnant or had eaten, drunk, exercised vigorously or smoked in the half hour before the measurement; a proportion of the remainder refused to have their blood pressure taken or failed to complete the measurements or measurements were not possible for technical reasons. Table 3 shows the numbers in each survey with valid BPs and their hypertensive categories.

Table 3: Hypertensive Categories of HSfE 2003 & 2004 Datasets & Merged 2003-2004 Dataset

	HSfE 2003		HSfE 2004		HSfE 2003+2004	
	Freq.	Percent	Freq.	Percent	Freq.	Percent

Refused/not applicable	5,569	38.1	3966	59.6	9,535	44.8
normotensive untreated	6,037	41.3	1,983	29.8	8,020	37.7
normotensive treated	579	4.0	180	2.7	759	3.57
hypertensive treated	699	4.8	171	2.6	870	4.09
hypertensive untreated	1,746	11.9	356	5.4	2,102	9.88
**Total with valid BP**	**9,061**	**61.9**	**2,690**	**40**	**11,751**	**55.2**
**Total**	**14,630**	**100**	**6,656**	**100**	**21,286**	**100**

2.2.2 Model construction: data issues

The choice of variables for original inclusion in the merged dataset included all those known to be high blood pressure risk factors. The variable names and labels are shown in Table 4 below. The HSfE dataset has a nested or hierarchical structure so three variables related to the sampling strata were included: area (sample point), cluster (stratification level), and hserial (serial number of household). These were used in the model to adjust for clustering of respondents. In the analysis variables cholest and hdlchol were combined to give a lipid ratio.

Table 5: Variables Included in Merged Dataset

Variable	Description	Source
ADDRESS5	Address number	Sample
ADTOT30	Adults: Total days/ 4week active 30min + moderate	Derived
ADTOT30C	Adults: Total days per week active 30min + moderate	Derived
AG16G10	(D) Age 16+ in ten year bands	Derived
AGE	Age last birthday	Individual
BIRTWT	Birth weight (kg)	Individual
BMI	BMI - inc unreliable measurements	Derived
BMIVAL	Valid BMI	Derived
BMIVG4	Valid BMI (grouped:<20,20-25,25-30,30+)	Derived
BP1	Doctor diagnosed high blood pressure (excluding pregnant)	Derived
BPMEDC	Whether taking drugs affecting blood pressure	Derived
BPMEDD	Whether taking drugs prescribed for blood pressure	Derived
CIGST1	Cigarette Smoking Status - Never/Ex-reg/Ex-occ/Current	Derived
COOKSALT	Salt added at when cooking? SC 16+	Individual
CVDDEF	Had cardiovascular condition	Derived
CVDDEF1	Had cardiovascular condition (excluding diabetes/high BP)	Derived
EQMEAN	EQ-5D social preference weight (mean)	Derived
ETHCIND	Ethnic group	Individual
EVERBP	Ever had high BP	Individual
GHQ12SCR	GHQ Score - 12 point scale	Derived
HBP140OM	Whether hypertensive: 140/90: all prescribed drugs for BP (Omron reading)	Derived
HHOLD	Household	Sample
HIBP1OM	Whether hypertensive: all prescribed drugs for BP (Omron reading)	Derived

Variable	Description	Source
HY140OM	Hypertensive catagories: 140/90: all prescribed drugs for BP (Omron reading)	Derived
HYPER1OM	Hypertensive catagories: all prescribed drugs for BP (Omron reading)	Derived
IMD2004	Index of multiple deprivation (SOA level)	Derived
LIMITILL	Limiting longstanding illness	Derived
MEDCINBP	Take medicines for high BP	Individual
NOBPCVD	Had CVD excludes those with high BP	Derived
NSSEC5	NS-SEC 5 variable classification (individual)	Derived
NSSEC8	NS-SEC 8 variable classification (individual)	Derived
OMDIAST	Omron Diastolic BP (mean 2nd/3rd) inc. invalid	Derived
OMDIAVAL	Omron Valid mean diastolic BP	Derived
OMMAP	Omron Mean arterial pressure (mean 2nd/3rd) inc. invalid	Derived
OMMAPVAL	Omron Valid mean arterial pressure	Derived
OMPULS	Omron Pulse pressure, systolic-diastolic inc. invalid	Derived
OMPULVAL	Omron Valid pulse pressure	Derived
OMSYST	Omron Systolic BP (mean 2nd/3rd) inc. invalid	Derived
OMSYSVAL	Omron Valid mean systolic BP	Derived
POINT	Sample point	Sample
SERIALH	Serial number of household	Hhold
SERIALI	Serial number of individual	Individual
SEX	Sex	Individual
STRATA	Stratification level	Individual
TABSALT	Salt added at table? SC 16+	Individual
TOPQUAL2	(D) Highest Educational Qualification - Students separate	Derived
TOPQUAL3	(D) Highest Educational Qualification	Derived

There was a problem with merging the variable for deprivation (Index of Multiple Deprivation 2004). The bandings of IMD scores were slightly different between the two years, but raw scores were not provided in the dataset so that it was necessary to assume identity (see Table 5 below).

Table 5: Index of Multiple Deprivation Banding

Rank	IMD Band	IMD		Number	Per Cent	Cum Per Cent
		HSfE 2003	HSfE 2004			
least	1	0.59-8.35	0.55-9.02	3,803	17.87	17.87
	2	8.35-13.72	9.03-14.14	3,573	16.79	34.65
	3	13.72-21.16	14.15-21.17	3,788	17.8	52.45
	4	21.16-34.21	21.18-33.52	4,551	21.38	73.83
most	5	34.21-86.36	33.53-85.69	5,571	26.17	100
			**Total**	**21,286**	**100**	

The Stata software package was used for analysis. All variables were recoded to drop negative values for estimation purposes (in HSfE various non-response categories are assigned negative values). The methodology applied was multinomial logistic regression with the “cluster” option (see above). For analysis of two categories as here, multinomial logistic regression is reduced to binomial logistic regression. However the reason for not using other logistic regression routines that take into account nested structures is that the other options available in Stata produced an estimation error, probably because of the small percentage of disease-positive respondents in the sample.

The modelling and estimation of the effects of interest was carried out using the mlogit command. The initial output consisted of two tables: one with the estimated regression coefficients, corresponding p-values and 95% confidence intervals, and another with the estimated odds ratios (exp(b)), which in the table appear as relative risk ratios (RRRs) and 95% confidence intervals. A positive sign of the estimated coefficient is associated with an increase in the odds of the outcome had angina or heart attack, and a negative sign is associated with a decrease in the odds. Since Prob (A) = Odds (A) / 1+ Odds (A), for uncommon outcomes such as high blood pressure, RRR can be assumed to be the same as the odds ratio (OR).

For categorical variables the effects are estimated relative to the reference category. Stata uses the first category as reference (baseline OR). Separate baseline odds were estimated for each gender, and also according to ethnicity, age band, area-based deprivation score etc. The model can be used to derive the prevalence ratios for high blood pressure for subjects with various combinations of risk factors in relation to baseline. The prevalence in each age group, gender, ethnic group, area of residence and level of deprivation, and smoking status category were derived from the odds, using the formula: prevalence = odds/(1 + odds).

2.2.3 Model construction: interactions between variables

Effect modification or interaction occurs if the effect of one exposure or risk factor on the outcome varies according to the level of another risk factor. We tested for interactions using a χ^2 test of heterogeneity (Mantel-Haenszel Odds Ratio) and Wald test. Where significant interaction was found, we performed a regression analysis of a generated interaction term. Where evidence of interaction was found across a range of strata, we introduced interaction terms or parameters into the final regression model. These allow the effect of one variable to be different in different categories of other variables. In Stata the xi command expands terms containing categorical variables into indicator (also called dummy) variable sets by creating new variables and estimates interactions and main effects.

2.2.4 Model construction: internal validation

Ideally the best prediction should result from utilising the most information in the regression model. However only a limited range of HSfE variable data is either available or can be estimated at the PCT level. We decided to validate the local model by comparing it, in terms of prediction, to a model including all available and significant HSfE variables. In addition, however, the amount of missing data affects the prediction of a model.

The local model used only used locally available data. In future body mass index (BMI) could be included on the basis that local synthetic estimates are now available and good local data from primary care may soon be. Local Limiting Longstanding Illness score data will be available locally from 2009 and could also be included later.

2.2.5 Model construction: external validation

An early external validation will be carried out by examining the association between PCT/LA level high blood pressure prevalence estimates and QOF registered prevalence. The regression-based model will also be validated against a prevalence model obtained by Bayesian strategies using WinBUGS. Finally, funding will be sought to undertake a validation of practice-based prevalence estimates against registered prevalence supplemented by active case finding.

2.3 The model

In developed countries, increasing age is by far the most important risk factor for high blood pressure, although this is not the case in non-developed agrarian societies. The age-related increase in hypertension prevalence appears after migration to urban environments. Table 6 shows the increasing numbers of treated and hypertensive untreated respondents in the dataset.

Table 6: Hypertension Category by Age Band

	Age Band							
	**16-24**	**25-34**	**35-44**	**45-54**	**55-64**	**65-74**	**75+**	**Total**
normotensive untreated	1,047	1,663	2,132	1,408	979	498	293	8,020
normotensive treated	1	7	45	123	239	224	120	759
hypertensive treated	0	6	31	93	225	265	250	870
hypertensive untreated	49	125	283	400	487	417	341	2,102
Total	1,097	1,801	2,491	2,024	1,930	1,404	1,004	11,751

2.3.1 Local model

The regression model for risk factors for hypertension in the “local” prevalence model is shown in Table 7. As expected ORs increase strikingly with increasing age in all models. In the prevalence predictions using coefficients (not shown in these tables) this results in age-related increases in prevalence which closely match the crude overall prevalences in Table 6. An age-sex interaction was found and so interaction terms for age and male sex were created. As expected a significant association was found between Black or Black British ethnicity and the outcome, and also with increasing deprivation.

Table 7: Odds ratios for variables included in “local” model

	RRR	Std. Err. of RRR	z	P>z	[95% Conf.	Interval]
Female Sex	1.000					
Male sex	3.197	1.010	3.680	0.000	1.721	5.937
Females Age 16-24	1.000					
Females Age 25-34	2.271	0.675	2.760	0.006	1.267	4.068
Females Age 35-44	5.201	1.431	5.990	0.000	3.034	8.917
Females Age 45-54	14.069	3.814	9.750	0.000	8.270	23.933
Females Age 55-64	39.730	10.720	13.650	0.000	23.413	67.420
Females Age 65-74	87.067	23.899	16.270	0.000	50.840	149.107
Females Age 75+	134.375	37.612	17.510	0.000	77.636	232.580
Interaction Male Sex & Age 16-24	1.000					
Interaction Male Sex & Age 25-34	0.684	0.251	-1.030	0.301	0.333	1.404
Interaction Male Sex & Age 35-44	0.572	0.191	-1.670	0.095	0.297	1.102
Interaction Male Sex & Age 45-54	0.574	0.190	-1.680	0.093	0.300	1.098
Interaction Male Sex & Age 55-64	0.370	0.121	-3.030	0.002	0.195	0.703
Interaction Male Sex & Age 65-74	0.265	0.089	-3.970	0.000	0.137	0.510
Interaction Male Sex & Age 75+	0.191	0.066	-4.800	0.000	0.097	0.376
White ethnic group	1.000					
Mixed ethnic group	0.920	0.256	-0.300	0.764	0.534	1.586
Black or Black British ethnic group	1.706	0.174	5.240	0.000	1.397	2.083
Asian or Asian British ethnic group	1.054	0.083	0.660	0.509	0.902	1.230
Any other ethnic group	0.972	0.155	-0.180	0.858	0.711	1.328
Index of multiple deprivation 0.59-8.35	1.000					
Index of multiple deprivation 8.35-13.72	0.996	0.073	-0.060	0.953	0.862	1.150
Index of multiple deprivation 13.73-21.16	1.159	0.085	2.010	0.044	1.004	1.339
Index of multiple deprivation 21.17-34.21	1.210	0.092	2.520	0.012	1.043	1.404
Index of multiple deprivation 34.22-86.36	1.346	0.105	3.810	0.000	1.156	1.568

2.3.1.1 Interactions between variables

We tested for interactions between variables. Significant interactions were found on Mantel-Haenszel Odds Ratio and Wald tests for sex and ethnicity, and sex and age. We therefore generated interaction terms for these variables in a regression model. Only age and sex showed a number of significant associations across strata, for the three oldest age-sex categories (p=0.000-0.003). We therefore included an interaction term in the final local model. This means that there are two separate effects of age on the outcome: one for males and another for females. The interaction terms shown represent the interaction between sex and age, and show the combined effect of being male and of a particular age group. The interaction is clearly demonstrated in the chart below of the prevalence of hypertension by age and sex. The prevalence of hypertension in females crosses over that in males in the older age groups.

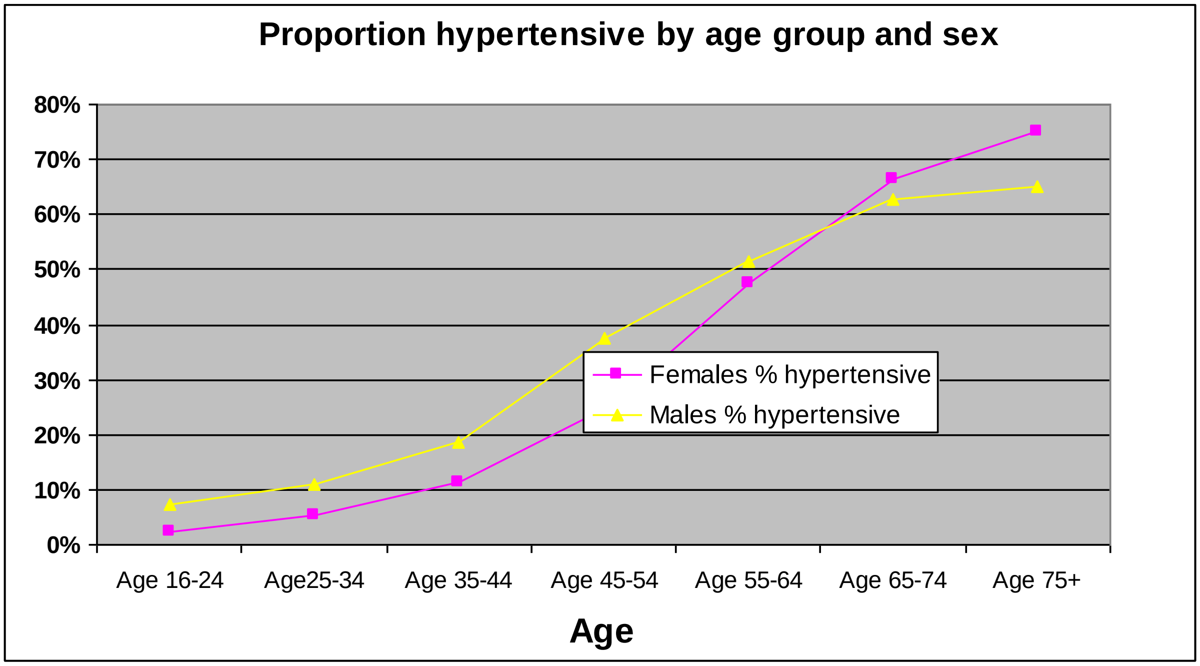

2.3.2 Complete model

The regression model for risk factors for high blood pressure in the "complete" model with BP drugs is shown in Table 8. Stepwise addition of variables in addition to age and sex was undertaken and only independent variables which were significant in this case were included in the final complete model. Variables which were eliminated through this process were:

- Cigarette Smoking Status - Never/Ex-reg/Ex-occ/Current
- Salt added at when cooking? SC 16+
- EQ-5D social preference weight (mean)
- GHQ Score - 12 point scale
- NS-SEC 5 variable classification (individual)

Table 9: Odds ratios for variables included in "complete" model

	RRR	Std. Err.	z	P>z	[95% Conf.	Interval]
Females	1.000					
Males	3.173	1.057	3.47	0.001	1.651	6.096
Females Age 16-24	1.000					
Females Age 25-34	1.834	0.584	1.9	0.057	0.982	3.423
Females Age 35-44	4.101	1.202	4.82	0	2.309	7.284
Females Age 45-54	9.937	2.881	7.92	0	5.630	17.540
Females Age 55-64	26.757	7.746	11.35	0	15.171	47.191
Females Age 65-74	54.697	16.224	13.49	0	30.583	97.824
Females Age 75+	99.816	30.945	14.85	0	54.363	183.270

	RRR	Std. Err.	z	P>z	[95% Conf.	Interval]
Interaction Male Sex & Age 16-24	1.000					
Interaction Male Sex & Age 25-34	0.787	0.306	-0.62	0.537	0.367	1.686
Interaction Male Sex & Age 35-44	0.589	0.208	-1.5	0.134	0.295	1.177
Interaction Male Sex & Age 45-54	0.600	0.210	-1.46	0.144	0.302	1.190
Interaction Male Sex & Age 55-64	0.375	0.130	-2.82	0.005	0.190	0.741
Interaction Male Sex & Age 65-74	0.286	0.102	-3.52	0	0.143	0.574
Interaction Male Sex & Age 75+	0.187	0.070	-4.49	0	0.090	0.389
**Ethnic Group**						
white	1.000					
mixed ethnic group	0.943	0.297	-0.19	0.853	0.510	1.747
black or black british	1.668	0.190	4.49	0	1.334	2.086
asian or asian british	1.092	0.095	1	0.315	0.920	1.296
any other group	1.095	0.197	0.51	0.612	0.770	1.558
**Total days/4 weeks active 30 mins + moderate +**						
none	1.000					
less than 1	0.762	0.066	-3.16	0.002	0.644	0.902
1 or 2 a week	0.832	0.063	-2.44	0.015	0.717	0.965
3 or 4 a week	0.750	0.069	-3.13	0.002	0.626	0.898
5 or more a week	0.694	0.052	-4.86	0	0.599	0.804
**Body mass index (BMI)**						
under 20	1.000					
20-25	1.860	0.318	3.64	0	1.331	2.600
25-30	2.996	0.506	6.5	0	2.153	4.171
Over 30	5.413	0.931	9.82	0	3.864	7.582
**Salt added at table**						
generally add salt to food without tasting it first	1.000					
taste the food, but then generally add salt	1.121	0.103	1.24	0.217	0.935	1.343
taste the food, but only occasionally add salt	1.078	0.089	0.91	0.365	0.917	1.268
rarely, or never, add salt at the table	1.225	0.093	2.66	0.008	1.055	1.423
**Ntiles of IMDscore**						
0.59->8.35 [least deprived]	1.000					
8.35->13.72	0.959	0.075	-0.54	0.591	0.822	1.118
13.72->21.16	1.105	0.087	1.26	0.206	0.946	1.291
21.16->34.21	1.118	0.094	1.32	0.186	0.948	1.318
34.21->86.36 [most deprived]	1.163	0.102	1.72	0.086	0.979	1.381
**Highest educational qualification separate**						
NVQ4/NVQ5/degree or equiv	1.000					
higher ed below degree	1.014	0.100	0.14	0.891	0.835	1.231
NVQ3/GCSE A level equiv	1.159	0.123	1.39	0.164	0.942	1.426
NVQ2/GCSE O level equiv	1.157	0.100	1.69	0.09	0.977	1.370
NVQ1/CSE other grade equiv	1.212	0.163	1.43	0.152	0.932	1.577

	RRR	Std. Err.	z	P>z	[95% Conf.	Interval]
foreign/other	1.440	0.195	2.7	0.007	1.105	1.878
no qualification	1.262	0.108	2.71	0.007	1.066	1.494
Full time student	1.363	0.184	2.29	0.022	1.046	1.776
**Limiting long-term illness**						
limiting long-term illness	1.000					
non limiting long-term illness	1.560	0.109	6.35	0	1.360	1.789
no long-term illness	0.882	0.055	-2.01	0.045	0.781	0.997

Although EQ-5D social preference weight (mean) and GHQ Score - 12 point scale were marginally significant when tested stepwise, they ceased to be so in the complete model and were excluded. In contrast, although ORs increase with increasing deprivation, the Index of Multiple Deprivation (SOA level) also ceased to be significant in the complete model but has been retained as it is included in the local model. ORs for IMD score from stepwise regression are shown in Table 8 below for comparison, and are similar to the local model. This could be explained by colinearity with highest educational qualification in the complete model.

Table 9: Odds ratios for Quintiles of IMD score (stepwise regression)

	RRR	Std. Err.	z	P>z	[95% Conf.	Interval]
0.59->8.35 [least deprived]	1.000					
8.35->13.72	0.999	0.073	-0.02	0.987	0.865	1.153
13.72->21.16	1.178	0.086	2.24	0.025	1.021	1.360
21.16->34.21	1.267	0.095	3.15	0.002	1.093	1.467
34.21->86.36 [most deprived]	1.458	0.109	5.05	0	1.260	1.688

As expected ORs increase markedly with increasing age in all models, with confidence intervals above 1 from age 35. Note that age group 16-24 has been defined as the baseline odds. There is a significantly lower overall OR for female sex and for most categories of increasing physical activity. This latter relationship has been described by numerous other studies and is one of the most important pathways through which physical activity reduces CVD risk. There is also a strong significant increase in ORs with increasing BMI. The result for salt added at the table is unexpected and counter-intuitive, with a significant increase in ORs for “rarely, or never, add salt at the table”. There was a good response rate for this question (14,478/21,286).

As in the local model ORs are significantly higher in the Black or Black British ethnic groups. There are some associations between measures of deprivation and educational level and the high blood pressure outcome.

2.4 Validation: area under Receiver Operating Characteristics curve (AUROC)

Receiver-operating characteristic (ROC) analysis was originally developed during World War II to analyze classification accuracy in differentiating signal from noise in radar detection. Recently, the methodology has been adapted to several clinical areas heavily dependent on screening and diagnostic tests, in particular, laboratory testing, epidemiology, radiology, and bioinformatics (7;8). ROC analysis is a useful tool for evaluating the performance of diagnostic tests and more generally for evaluating the accuracy of a statistical model (e.g. logistic regression, linear discriminant analysis) that classifies subjects into one of two categories, diseased or non-diseased, as in this model (9). Its function as a simple graphical tool for displaying the accuracy of a medical diagnostic test is one of the most well-known applications of ROC curve analysis.

An ROC curve is a plot of sensitivity on the *y* axis against (1-specificity) on the *x* axis for varying values of the threshold t. The 45° diagonal line connecting (0,0) to (1,1) is the ROC curve corresponding to random chance. The ROC curve for the gold standard is the line connecting (0,0) to (0,1) and (0,1) to (1,1). Generally, ROC curves lie between these two extremes. The area under the ROC curve is a summary measure that essentially averages diagnostic accuracy across the spectrum of test values. The area under the curve (AUC) is an overall summary of diagnostic accuracy. AUC equals 0.5 when the ROC curve corresponds to random chance and 1.0 for perfect accuracy. On rare occasions, the estimated AUC is <0.5, indicating that the test does worse than chance.

AUROCs for the local and complete models tested above were estimated using Stata10. These are shown in the charts below. If both sensitivity and specificity are of importance in a high blood pressure model, the optimal threshold of t would be 0.75, where sensitivity and specificity equal 0.77. The local model, with an AUROC of 0.8071 exceeds this level, although the complete model has even better performance, with an AUROC of 0.8304.

Chart 1: ROC Curve for local hypertension model

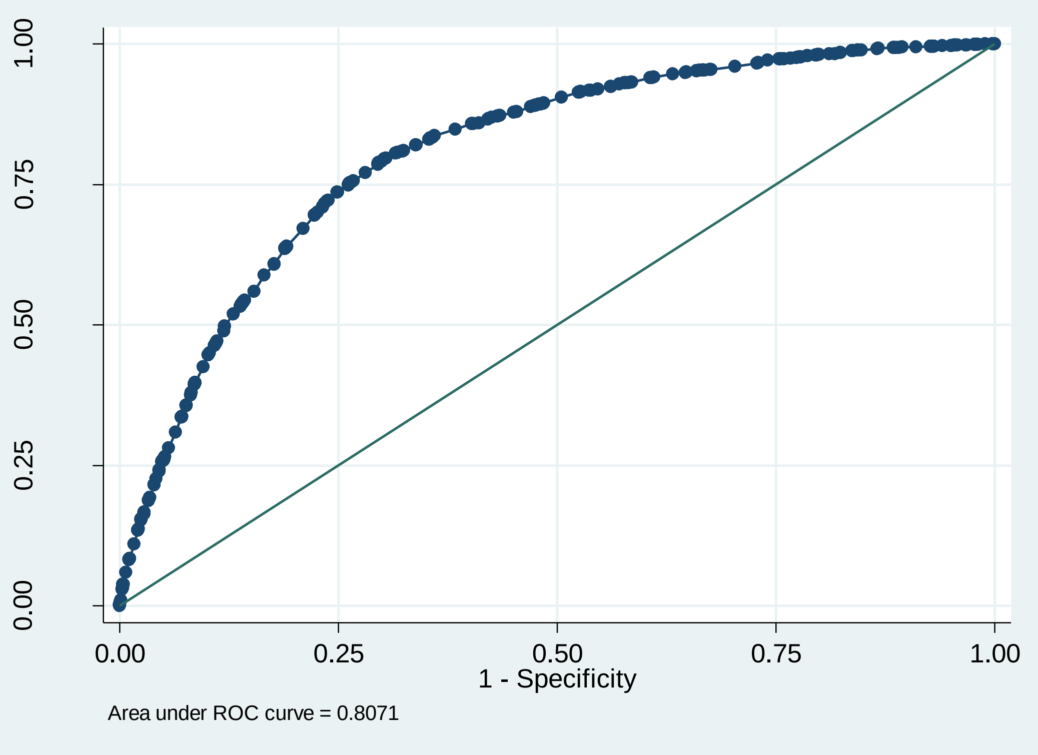

Chart 2: ROC Curve for complete hypertension model

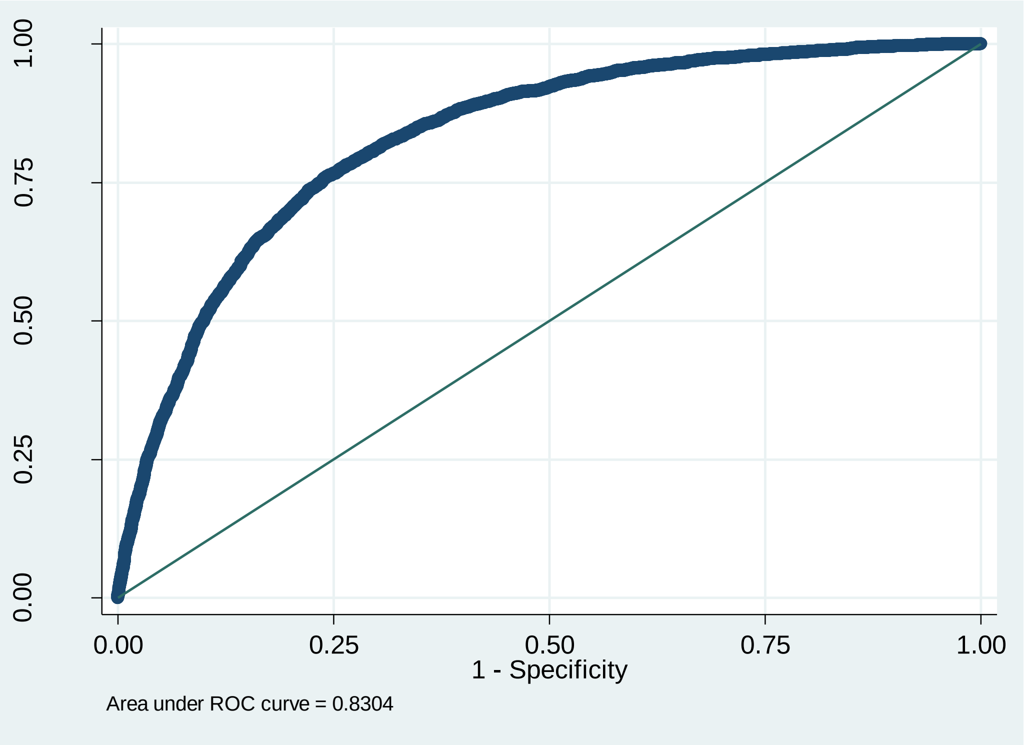

2.4.1 Validation: model prediction

Another method of assessing performance is to use the regression model to predict the response for each subject. These predictions are called fitted values. The difference between the fitted and the observed values are called residuals. These can then be tabulated against the observed presence of high blood pressure to assess "misclassification" by each model (in shaded cells). The results for the local and complete models are shown in the table below. The complete model shows slightly lower misclassification but this is minimal in view of the lower overall numbers in this model.

Table: 10 Comparison of observed: predicted hypertension- local model

		Predicted		
		**Not_hypertensive**	**Hypertensive**	**Total**
**Observed**	**Not_hypertensive**	6,886	1,129	8,015
	**Hypertensive**	1,717	2,012	3,729
	**Total**	8,603	3,141	11,744

Table: 11 Comparison of observed: predicted hypertension- complete model

		Predicted		
		**Not_hypertensive**	**Hypertensive**	**Total**
**Observed**	**Not_hypertensive**	6,446	938	7,384
	**Hypertensive**	1,424	1,878	3,302
	**Total**	7,870	2,816	10,686

3 Application of the Model

The local model (which includes only those variables that are available at population level i.e. age, sex, ethnicity, smoking status and deprivation score) has been applied to Local Authorities to create prevalence estimates of hypertension in those aged 16+ for 2005–2020. Models for PCTs have been created for 2006-2020.

3.1 Assumptions of the modelled estimates

It is assumed that:

- the prevalence of hypertension in those aged under 16 is negligible.

3.2 Input data

3.2.1 Populations

3.2.1.1 Local Authorities

The hypertension prevalence model uses ONS 2005 mid-year population estimates by ethnic group, age and sex. ONS publishes the data by broad age band (10), but supplied full quinary age-band data to APHO for the prevalence modelling project. Five ethnic groups were used: white, black, Asian, mixed and other.

3.2.1.2 Primary Care Trusts

The hypertension prevalence model uses ONS 2006 mid-year population estimates by ethnic group, age and sex. ONS publishes the data by broad age band (10), but supplied full quinary age-band data to APHO for the prevalence modelling project. Five ethnic groups were used: white, black, Asian, mixed and other.

3.2.1.3 Population projections

In order to calculated estimate prevalence of hypertension in the future, population projections were incorporated into the model. ONS has not published population projections by ethnic group, so the 2005 (LA) or 2006 (PCT) distribution of ethnic groups was used to generate population estimates to 2020.

For 2006 and 2007 LA population projections, the distribution of ethnic groups in 2005 was applied to ONS mid-year population estimates by quinary age band. For 2007 PCT population projections, the distribution of ethnic groups in 2006 was applied to ONS mid-year population estimates by quinary age band.

For 2008 and 2009 the distribution of ethnic groups in 2005 (LA) or 2006 (PCT) was applied to ONS 2006-based population projections by quinary age band for LAs and PCTs.

For 2010, 2015 and 2020 the distribution of ethnic groups in 2005 (LA) or 2006 (PCT) was lagged by 5, 10 and 15 years respectively and combined with ONS 2006-based population projections by quinary age band for LAs and PCTs. For example, the ethnic proportions for the age band 40-45 in 2005 were applied to the population aged 50-55 in 2015. With the exception of this 'ageing' of the ethnic population, no other changes to the distribution of ethnic groups were considered.

3.2.2 Deprivation

Deprivation scores are taken from IMD 2004 (11). Deprivation scores for PCTs were calculated by taking a population weighted average of the scores for each MSOA (which in turn were calculated by taking a weighted average of the IMD2004 scores of each LSOA) within the PCT.

Five deprivation categories are used in the model. Note that these categories are based on quintiles of IMD score at LSOA level. When the cut-offs are applied to larger geographies (LA or PCT) there is not an even distribution across the categories.

4 References

1. Faculty of Public Health hypertension prevalence model http://www.fphm.org.uk/resources/AtoZ/toolkit_hypertension/tools.asp
2. Lawes CM, Hoorn SV, Rodgers A. Global burden of blood-pressure-related disease, 2001. The Lancet 2008; 371(9623):1513-1518.
3. Wolf-Maier K, Cooper RS, Banegas JR, Giampaoli S, Hense HW, Joffres M et al. Hypertension Prevalence and Blood Pressure Levels in 6 European Countries, Canada, and the United States. JAMA 2003; 289(18):2363-2369.
4. Hajat C, Tilling K, Stewart JA, Lemic-Stojcevic N, Wolfe CDA. Ethnic Differences in Risk Factors for Ischemic Stroke: A European Case-Control Study. Stroke 2004; 35(7):1562-1567.
5. Lawes CMM, Bennett DA, Feigin VL, Rodgers A. Blood Pressure and Stroke: An Overview of Published Reviews. Stroke 2004; 35(3):776-785.
6. National Institute for Health and Clinical Excellence. Hypertension: Management of hypertension in adults in primary care. National Institute for Health and Clinical Excellence [2006 [cited 2008 May 13]; **NICE clinical guideline 34** Available from: URL:http://www.nice.org.uk/nicemedia/pdf/CG034NICEguideline.doc
7. Hanley JA, McNeil BJ. The meaning and use of the area under a receiver operating characteristic (ROC) curve. Radiology 1982; 143(1):29-36.
8. Hanley JA, McNeil BJ. A method of comparing the areas under receiver operating characteristic curves derived from the same cases. Radiology 1983; 148(3):839-843.
9. Zou KH, O'Malley AJ, Mauri L. Receiver-Operating Characteristic Analysis for Evaluating Diagnostic Tests and Predictive Models. Circulation 2007; 115(5):654-657.
10. ONS population estimates by ethnic group http://www.statistics.gov.uk/STATBASE/Product.asp?vlnk=14238
11. IMD 2004 http://www.communities.gov.uk/archived/general-content/communities/indicesofdeprivation/216309/
