# Supplementary material for: Variations in cardiovascular disease under-diagnosis in England: national cross-sectional spatial analysis
Source: BMC Cardiovasc Disord. 2011 Mar 17;11:12. doi: 10.1186/1471-2261-11-12 (PMC3070686; doi:10.1186/1471-2261-11-12)
Supplement: Additional file 3 — Stroke prevalence modelling briefing document v2. This document describes how the hypertension prevalence model was developed from Health Survey for England data and how the model was applied to local population data. [file 1471-2261-11-12-S3.PDF]

Stroke Prevalence Modelling Briefing Document

Hannah Walford, ERPHO

Michael Soljak and Azeem Majeed, Department of Primary Care and Social Medicine & Statistical Advisory Service, Imperial College London

November 2008

This briefing document explains how the stroke prevalence model has been developed and applied. It accompanies the stroke prevalence estimates released in October 2008, and also gives details of the adjustment made for haemorrhagic stroke at the request of the Healthcare Commission.

The model follows a similar methodology to the CHD and hypertension models recently published by ERPHO. The model was developed by Michael Soljak of the Department of Primary Care and Social Medicine, Imperial College, London on behalf of APHO.

Contents

1 Development of Stroke Prevalence Model 3
1.1 Background 3
1.1.1 Prevalence of stroke 3
1.1.1 Incidence and outcomes of stroke 5
1.1.2 Previous stroke prevalence modelling 6
1.2 Methods 6
1.2.1 Data Sources 6
1.2.2 Model construction: data issues 7
1.2.3 Model construction: interactions between variables 8
1.2.4 Model construction: validation 8
1.3 The Model 9
1.3.1 Sensitivity/Specificity: ROC Curve 9
1.3.2 Local Model 11
1.3.3 Complete model 12
1.3.4 Prediction 14
2 Application of the model 14
2.1 Assumptions of the modelled estimates 14
2.2 Input data 15
2.2.1 Populations 15
2.2.2 Smoking status 15
2.2.3 Deprivation 17
2.3 Adjustment for haemorrhagic stroke 17
3 References 18

1 Development of Stroke Prevalence Model

1.1 Background

1.1.1 Prevalence of stroke

A report from WHO estimated stroke incidence and prevalence for each European country from routine mortality statistics (1). Rates from studies that met the 'ideal' criteria were compared with WHO's estimates. Forty-four incidence studies and twelve prevalence studies were identified. WHO stroke estimates were in good agreement with results from 'ideal' stroke population studies. According to the WHO estimates, the number of stroke events in these selected countries is likely to increase from 1.1 million per year in 2000 to more than 1.5 million per year in 2025 solely because of the demographic changes.

In men, the lowest stroke prevalence rates are estimated for Cyprus, Lithuania, Poland, and Slovakia, whilst the highest rates are estimated for Czech Republic, Greece, Portugal, and Slovenia. In women, low prevalence rates are estimated for Cyprus, France, Lithuania, Poland, and Slovakia, whilst high prevalence rates are estimated for Czech Republic, Greece, Hungary, and Portugal. Table 1 below shows the WHO prevalence estimates for the UK.

Table 1: UK stroke prevalence rates, estimates from the World Health Organization, men and women per 1000 population (1)

Age	Men	Women
25–34	0.33	0.93
35–44	0.63	1.77
45–54	4.55	9.52
55–64	8.47	20.21
65–74	20.62	50.16
75–84	39.11	79.18
85+	56.39	93.15

Another international review of stroke epidemiology found nine high quality prevalence studies (2). Overall, 8,788 strokes were reported, with age-specific prevalence increasing with age. The age-standardised prevalence for people aged 65 years or more ranged from 46.1 to 73.3 per 1000 population, but ranged from 58.8 to 92.6 per 1000 population for men, and from 32.2 to 61.2 per 1000 population for women. The small variation in age-specific and age-standardised prevalence of stroke across the populations (five to ten per 1000) was consistent with the geographical similarity in stroke incidence and case-fatality.

A systematic review of stroke epidemiology in South America found stroke prevalence rates ranging from 1.74 to 6.51 per 1000, and annual incidence rates from 0.35 to 1.83 per 1000 (3). Community-

based studies showed crude stroke prevalence rates ranging from 1.74 to 6.51 per 1000 and annual incidence rates from 0.35 to 1.83 per 1000. In a population survey in Berlin of a total of 75,720 households (28,090 persons responded), a total of 4.5% reported a physician-diagnosed stroke (women 4.3%; men 4.9%) (4). Combining reported stroke history with reported impaired vision and/or articulation problems, the prevalence of stroke increased to 7.6% (men 8.4%; women 7.2%).

There have been three recent published UK prevalence studies. A point prevalence study using postal questionnaires (n=18,000) in northern England found that prevalence increased with age and, apart from the very elderly, males had a higher prevalence than females (5). Overall prevalence was found to be 46.8/1,000 (95% CI 42.5, 51.6). Full recovery from stroke was reported by 23% of respondents. Cognitive impairments (33%), problems with lower limbs (33% for right leg; 27% for left leg) and speech difficulties (27%) were the most common residual impairments.

In another UK prospective study all incident cases of neurological disorders were ascertained in an unselected urban population based in 13 general practices in the London area (6). A population of 100,230 patients registered with the practices was followed prospectively for the onset of neurological disorders, using multiple methods of case finding. Lifetime prevalence rates, expressed as rate per 1,000 persons with 95% CI, were: completed stroke 9/1,000 (CI: 8, 11); and for transient ischaemic attacks 5/1000 (CI: 4, 6).

A two-stage point prevalence study in Newcastle used a valid screening questionnaire to identify stroke survivors from a stratified sample (7). This was followed by assessment of stroke patients with scales of disability and handicap. The overall prevalence of stroke was found to be 17.5/1,000 (95% CI 17.0-18.0). The prevalence of stroke-associated dependence was 11.7/1,000 (95% CI 11.3-12.1).

Tables 2 and 3 show percentage prevalences of stroke in ethnic minority groups and the general population, and in various age groups, for the Health Survey for England (HSfE) 2004 (8). Informants are classified as having IHD or stroke if they reported having angina, a heart attack or a stroke, confirmed by a doctor. In summary, prevalence is estimated in these studies to be between one and five per cent, with the HSfE estimate falling midway in this range.

Table 2: Prevalence of stroke, by minority ethnic group and sex, aged 16 and over 2004

	Black Caribbean	Black African	Indian	Pakistani	Bangladeshi	Chinese	Irish	General population
**Men**								
**Observed %**	3.4	-	1.1	1.8	1.8	0.7	4.5	2.4
**Standardised risk ratios**	1.26	0.00	0.59	1.06	2.05	0.71	1.98	1
**Standard error of the ratio**	0.55	0.00	0.25	0.46	1.02	0.43	0.70	
**Women**								
**Observed %**	1.8	0.5	1.2	1.7	1.8	0.4	2.7	2.2
**Standardised risk ratios**	1.31	0.69	0.72	2.25	2.73	0.22	1.20	1
**Standard error of the ratio**	0.42	0.43	0.27	0.86	1.02	0.17	0.44	

Table 3: Prevalence of stroke, by age within minority ethnic group and sex, aged 16 and over 2004

	**Men**				**Women**			
	**16-34**	**35-54**	**55+**	**All**	**16-34**	**35-54**	**55+**	**All**
	%	%	%	%	%	%	%	
**Black Caribbean**	-	-	11.5	3.4	-	1.1	5.6	1.8
**Black African**	-	-	[-]	-	0.4	0.4	[1.5]	0.5
**Indian**	-	-	5.2	1.1	-	1.0	4.2	1.2
**Pakistani**	-	1.1	9.6	1.8	0.2	0.9	10.1	1.7
**Bangladeshi**	-	1.9	[9.2]	1.8	0.3	1.6	11.9	1.8
**Chinese**	-	0.8	2.2	0.7	-	0.5	0.8	0.4
**Irish**	-	2.2	9.4	4.5	0.9	0.6	6.3	2.7
**General population (2003)**	0.3	0.7	6.4	2.4	0.3	0.7	5.2	2.2

Source: Health Survey for England 2004

The GP practice ascertainment study in South London may therefore have underestimated prevalence because practices were unaware of a proportion of stroke victims. This is consistent with 2006-7 Quality & Outcomes Framework (QOF) data, which shows an observed/registered crude prevalence (whole population denominator) of only 1.61 per cent.

1.1.1 Incidence and outcomes of stroke

In the review quoted above, eight population-based studies assessing secular trends in stroke incidence in a given population were identified (2). Although the studies covered different periods, several common themes were evident. Most of the studies showed a decline in stroke incidence, through to the late 1970s or early 1980s. In several studies, however, this decline seemed to have reached a plateau or

even reversed in the late 1980s and early 1990s. Of the few population-based studies that reported time-trend data for stroke mortality, the consistent finding was of a decrease in rates from the 1970s through to the 1990s. About half to three-quarters of cases had stroke-related disability.

In South London, stroke incidence decreased over a 10-year time period (9). The greatest decline in incidence was observed in black women, but ethnic group disparities still exist, indicating a higher stroke risk in black people compared to white people. Total stroke incidence was higher in blacks compared to whites (IRR 1.27, 95% CI 1.10-1.46 in men; IRR 1.29, 95% CI 1.11 to 1.50 in women), but the black-white gap reduced during the 10-year time period (IRR 1.43, 95% CI 1.13-1.82 in 1995 to 1996 to 1.18, 95% CI 0.93-1.49 in 2003 to 2004). Age- and sex-adjusted IRRs for haemorrhagic stroke were higher in Black Africans (IRR, 2.80; 95% CI, 2.00 to 3.91) than in Black Caribbeans (IRR, 1.46; 95% CI, 1.07 to 1.99) which could be explained by pre-stroke hypertension being more common among young blacks (10).

The South London study also used capture-recapture models including covariates to estimate incidence of stroke (11). This suggested that the stroke register was 88% complete. Adjusting for under-ascertainment increased the estimated incidence from 1.31 (95% CI : 1.21-1.42) to 1.49 (95% CI : 0.38-2.60) per 1000.

1.1.2 Previous stroke prevalence modelling

There has apparently been very little previous published modelling of stroke prevalence. A simple prevalence model was included in the ASSET stroke package released by the Department of Health, but it is no longer available. It used only South London study data.

1.2 Methods

1.2.1 Data Sources

The stroke model described here uses data from the 2003 and 2004 Health Surveys for England (HSfE). The 2003 Survey contains data on a total of 18,553 individuals. The 2004 data consists of two individual level files of which one (10,114 records) contains data for all individuals in the Ethnic Boost Sample and informants in the General Population Sample who were of the specified ethnic groups in co-operating households who gave a full interview. It contains information from the household questionnaire, main individual schedule, self-completions and the nurse visit (where one occurred). Data on under 16s was dropped from both these raw data files for the regression modelling.

Because the HSfE showed that stroke prevalence varies with ethnicity (12), it was necessary to use a sample containing data from a large number of ethnic minority respondents. The HSfE 2004 was the last Survey to include an ethnic minority boost, and the boost sample was used for the modelling. However there were relatively small numbers of Whites in the HSfE 2004 sample, and BP was not measured for most respondents in the 2004 general population sample, presumably to save resources for the boost itself. The HSfE 2004 boost sample was therefore merged with the HSfE 2003 data, which measured BP and which was the year with the largest number of identical variables. Table 4 shows the ethnic group

breakdown of the two samples. Note that it was necessary to collapse two of the HSfE 2003 ethnic group variables in order to use the same classification as HSfE 2004.

Table 4: Ethnic Group Breakdown of HSfE 2003 & 2004 Datasets & Merged 2003-2004 Dataset

	**HSfE 2003**		**HSfE 2004**		**HSfE 2003+2004**	
	**Freq.**	**Percent**	**Freq.**	**Percent**	**Freq.**	**Percent**
white	13,445	92.11	1,130	16.98	14,575	68.64
mixed ethnic group	86	0.59	222	3.34	308	1.45
black or black british	308	2.11	1,683	25.29	1,991	9.38
asian or asian british	594	4.07	3,131	47.04	3,725	17.54
any other group	163	1.12	472	7.09	635	2.99
**Total**	**14,596**	**100**	**6,638**	**100**	**21,234**	**100**

NB excludes no answer/refused/don't know

1.2.2 Model construction: data issues

The choice of variables for original inclusion in the merged dataset included all those known to be stroke risk factors. The variable names and labels are shown in Table 3 below. The HSfE dataset has a nested or hierarchical structure so three variables related to the sampling strata were included: area (sample point), cluster (stratification level), and hserial (serial number of household). These were used in the model to adjust for clustering of respondents. In the analysis variables cholest and hdlchol were combined to give a lipid ratio.

There was a problem with merging the variable for deprivation (Index of Multiple Deprivation 2004). The bandings of IMD scores were slightly different between the two years, but raw scores were not provided in the dataset so that it was necessary to assume identity (see Table 5 below).

Table 5: Index of Multiple Deprivation Banding

**Rank**	**IMD Band**	**IMD**		**Number**	**Per Cent**	**Cum Per Cent**
		**HSfE 2003**	**HSfE 2004**			
least	1	0.59-8.35	0.55-9.02	3,803	17.87	17.87
	2	8.35-13.72	9.03-14.14	3,573	16.79	34.65
	3	13.72-21.16	14.15-21.17	3,788	17.8	52.45
	4	21.16-34.21	21.18-33.52	4,551	21.38	73.83
most	5	34.21-86.36	33.53-85.69	5,571	26.17	100
			**Total**	**21,286**	**100**	

The Stata software package was used for analysis. All variables were recoded to drop negative values for estimation purposes (in HSfE various non-reponse categories are assigned negative values). The methodology applied was multinomial logistic regression with the “cluster” option (see above). For analysis of two categories as here, multinomial logistic regression is reduced to binomial logistic regression. However the reason for not using other logistic regression routines that take into account nested structures is that the other options available in Stata produced an estimation error, probably because of the small percentage of disease-positive respondents in the sample.

The modelling and estimation of the effects of interest was carried out using the mlogit command. The initial output consisted of two tables: one with the estimated regression coefficients, corresponding p-values and 95% confidence intervals, and another with the estimated odds ratios (exp(b)), which in the table appear as relative risk ratios (RRRs) and 95% confidence intervals. A positive sign of the estimated coefficient is associated with an increase in the odds of the outcome had a history of stroke, and a negative sign is associated with a decrease in the odds. Since Prob (A) = Odds (A) / 1+ Odds (A), for uncommon outcomes such as stroke, RRR can be assumed to be the same as the odds ratio (OR).

For categorical variables the effects are estimated relative to the reference category. Stata uses the first category as reference (baseline OR). Separate baseline odds were estimated for each gender, and also according to ethnicity, age band, area-based deprivation score etc. The model can be used to derive the prevalence ratios for stroke for subjects with various combinations of risk factors in relation to baseline. The prevalence in each age group, gender, ethnic group, area of residence and level of deprivation, and smoking status category were derived from the odds, using the formula: prevalence = odds/(1 + odds).

1.2.3 Model construction: interactions between variables

Effect modification or interaction occurs if the effect of one exposure or risk factor on the outcome varies according to the level of another risk factor. This can be tested using a χ^2 test of heterogeneity, or by introducing interaction terms or parameters into the regression model. These allow the effect of one variable to be different in different categories of other variables. In Stata the xi command expands terms containing categorical variables into indicator (also called dummy) variable sets by creating new variables and estimates interactions and main effects.

1.2.4 Model construction: validation

Ideally the best prediction should result from utilising the most information in the regression model. However only a limited range of HSfE variable data is either available or can be estimated at the PCT level. We decided to validate the local model by comparing it, in terms of prediction, to a model including all available HSfE variables. In addition, however, the amount of missing data affects the prediction of a model. For example, in the complete HSfE variables the largest proportion of missing data occurred in those variables related to particular drug treatment for high blood pressure. Some clinical tests also have a high proportion of missing values. Hence the “complete” model included the

complete list of variables, including differing definitions of high blood pressure (history of high blood pressure, on drugs for high blood pressure, or clinically measured hypertension >140/90 at the time of the Survey).

The local model only used locally available data. We included smoking on the basis that local synthetic estimates are now available. Local GHQ-12 or Limiting Longstanding Illness score data will be available locally from 2009 and could be included later.

1.3 The Model

Table 7 shows crude stroke prevalence in the HSfE 2003-4 merged dataset.

Table 7: crude stroke prevalence in 2003-4 dataset

	Age 16-24	Age 25-34	Age 35-44	Age 45-54	Age 55-64	Age 65-74	Age 75+	Total
**No stroke**	2,498	3,850	4,429	3,435	2,972	2,131	1,476	20,791
%	99.84	99.72	99.48	98.82	97.31	93.51	88.86	97.68
**Stroke**	4	11	23	41	82	148	185	494
%	0.16	0.28	0.52	1.18	2.69	6.49	11.14	2.32
**Total**	2,502	3,861	4,452	3,476	3,054	2,279	1,661	21,285

1.3.1 Sensitivity/Specificity: ROC Curve

Sensitivity and specificity were calculated using the area under the receiver operating characteristics (ROC) curve. ROC analysis was originally developed during World War II to analyse classification accuracy in differentiating signal from noise in radar detection. Recently, the methodology has been adapted to several clinical areas heavily dependent on screening and diagnostic tests, in particular, laboratory testing, epidemiology, radiology, and bioinformatics (13;14). ROC analysis is a useful tool for evaluating the performance of diagnostic tests and more generally for evaluating the accuracy of a statistical model (e.g. logistic regression, linear discriminant analysis) that classifies subjects into one of two categories, diseased or non-diseased, as in this model (15). Its function as a simple graphical tool for displaying the accuracy of a medical diagnostic test is one of the most well-known applications of ROC curve analysis.

A ROC curve is a plot of sensitivity on the *y* axis against (1-specificity) on the *x* axis for varying values of the threshold t. The 45° diagonal line connecting (0,0) to (1,1) is the ROC curve corresponding to random chance. The ROC curve for the gold standard is the line connecting (0,0) to (0,1) and (0,1) to (1,1). Generally, ROC curves lie between these two extremes. The area under the ROC curve is a summary measure that essentially averages diagnostic accuracy across the spectrum of test values. The area under the curve (AUC) is an overall summary of diagnostic accuracy. Area under ROC curve equals 0.5 when the curve corresponds to random chance and 1.0 for perfect accuracy. On rare occasions, the estimated area under the curve is less than 0.5, indicating that the test does worse than chance. If both sensitivity and specificity are of importance in a model, the optimal threshold would be 0.75.

Area under ROC curves for the local and complete models tested were estimated using Stata10. These are shown in the table and chart below. The local model, with an AUROC of 0.834 exceeds the optimal threchold, although the complete model using a history of high blood pressure has even better performance, with an AUROC of 0.874. This data would also be easiest to estimate at a local level (possibly using the hypertension model). The table illustrates well the impact of missing data on area under ROC curve. Exact binomial and Hanley confidence intervals were calculated. Hanley specifies that the standard error for the area under the ROC curve be calculated using the method suggested by Hanley and McNeil. Otherwise, standard errors are obtained as suggested by DeLong and Clarke-Pearson.

Table 8: Comparison of area under ROC curves for differing models

Model/BP Variable(s)	ROC Test	Obs (n)	Area under ROC	Std. Err.	Lower 95% C. I.	Upper 95% C. I.
Local	Binomial Exact	21,136	0.834	0.009	0.829	0.839
	Hanley-Asymp Normal	21,136	0.834	0.009	0.818	0.851
Complete, 3 BP variables	Binomial Exact	4,915	0.828	0.015	0.817	0.839
	Hanley-Asymp Normal	4,915	0.828	0.015	0.799	0.858
Complete, on BP meds	Binomial Exact	6,003	0.819	0.014	0.809	0.829
	Hanley-Asymp Normal	6,003	0.819	0.014	0.793	0.846
Complete, ever had high BP	Binomial Exact	16,669	0.874	0.010	0.869	0.879
	Hanley-Asymp Normal	16,669	0.874	0.010	0.854	0.894
Complete, hypertensive >140/90 Omron	Binomial Exact	10,227	0.868	0.013	0.861	0.875
	Hanley-Asymp Normal	10,227	0.868	0.013	0.843	0.894

Figure 1: Comparison of area under ROC curves for differing models

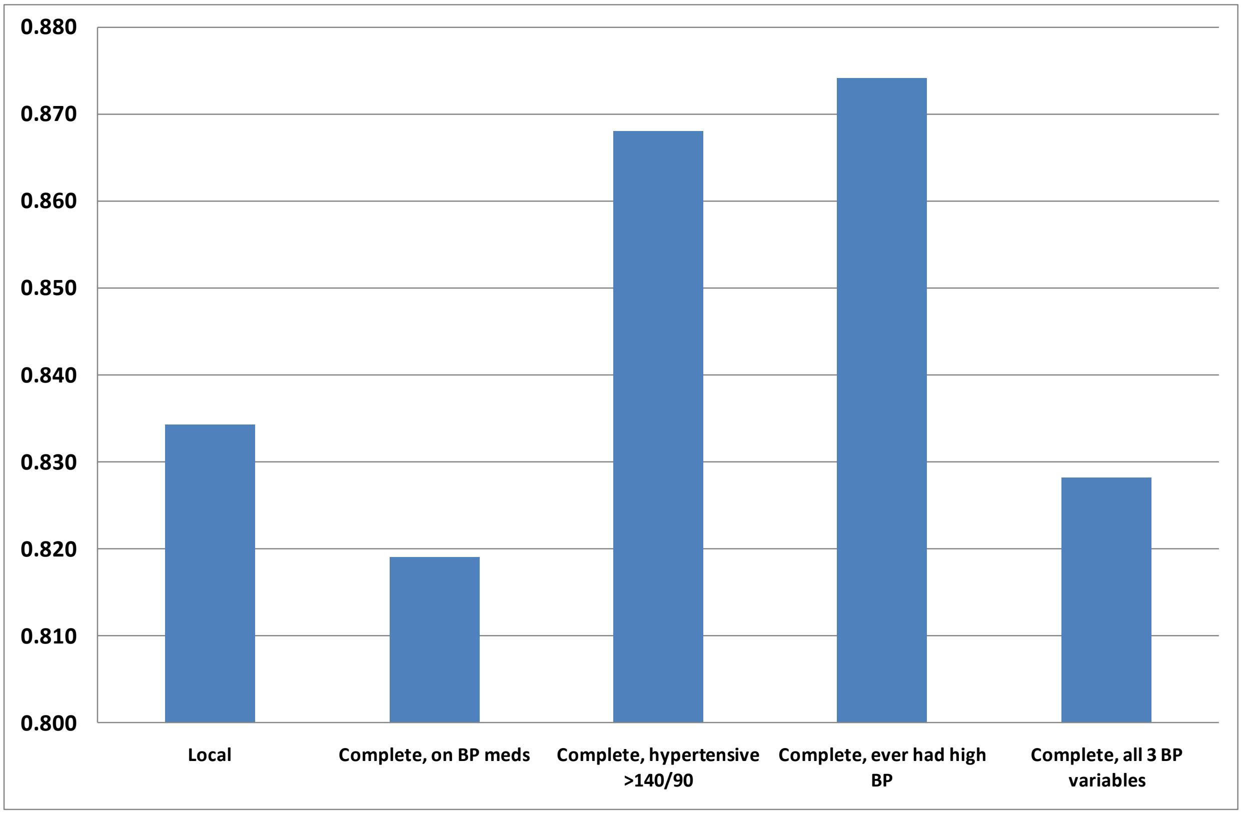

1.3.2 Local Model

Odds ratios, p values and confidence intervals are generally similar to the more complete models. Variables which should be included as inputs to the local model are highlighted. Unfortunately, however, local synthetic estimates do not have separate categories for occasional/regular smokers.

Table 9: Odds Ratios, Local Model

Column1	RRR	Std. Err.	z	P>z	[95% Conf.	Interval]
**Female**	1.000					
**Male**	1.144	0.111	1.39	0.166	0.946	1.383
**Age 16-24**	1.000					
**Age 25-34**	1.712	0.999	0.92	0.357	0.545	5.375
**Age 35-44**	3.254	1.764	2.18	0.03	1.124	9.418
**Age 45-54**	7.329	3.852	3.79	0	2.616	20.532
**Age 55-64**	17.652	9.092	5.57	0	6.432	48.443
**Age 65-74**	43.780	22.307	7.42	0	16.128	118.846
**Age 75+**	82.058	41.833	8.65	0	30.212	222.878

Column1	RRR	Std. Err.	z	P>z	[95% Conf.	Interval]
Never smoker	1.000					
Used to smoke occasionally	0.947	0.212	-0.24	0.809	0.611	1.469
Used to smoke regularly	1.353	0.150	2.72	0.006	1.088	1.682
Current smoker	1.389	0.189	2.41	0.016	1.063	1.814
IMD 2004 0.59->8.35 [least deprived]	1.000					
IMD 2004 8.35->13.72	1.233	0.199	1.3	0.193	0.899	1.692
IMD 2004 13.72->21.16	1.236	0.199	1.32	0.188	0.902	1.694
IMD 2004 21.16->34.21	1.397	0.217	2.15	0.032	1.030	1.895
IMD 2004 34.21->86.36 [most deprived]	2.009	0.291	4.83	0	1.513	2.668

1.3.3 Complete model

Table 10 shows odds ratios and other statistics for the complete model, using history of high blood pressure as the variable with the highest area under ROC curve. Included variables were selected by reverse stepwise selection using repeated likelihood ratios and Wald tests as recommended by Kirkwood and Sterne. A threshold probability value of 0.2 was used (see Table 11).

Table 10: Odds Ratios, Complete Model

	RRR	Std. Err.	z	P>z	[95% Conf.	Interval]
Age 16-24	1.000					
Age 25-34	1.434	0.853	0.61	0.544	0.447	4.599
Age 35-44	1.785	1.003	1.03	0.303	0.593	5.369
Age 45-54	2.907	1.591	1.95	0.051	0.995	8.499
Age 55-64	5.724	3.040	3.29	0.001	2.022	16.207
Age 65-74	15.196	8.026	5.15	0	5.397	42.787
Age 75+	21.734	11.547	5.8	0	7.672	61.571
Limiting long-term illness						
Non limiting long-term illness	0.387	0.065	-5.6	0	0.278	0.539
No long-term illness	0.291	0.051	-7.1	0	0.207	0.410
IMD 2004 0.59->8.35 [least deprived]						
IMD 2004 8.35->13.72	1.305	0.250	1.39	0.165	0.896	1.900
IMD 2004 13.72->21.16	1.008	0.202	0.04	0.968	0.680	1.494
IMD 2004 21.16->34.21	1.012	0.202	0.06	0.951	0.685	1.497
IMD 2004 34.21->86.36 [most deprived]	1.322	0.259	1.42	0.154	0.900	1.942
Never had high BP						
Ever had high BP	2.767	0.385	7.31	0	2.106	3.635

	RRR	Std. Err.	z	P>z	[95% Conf.	Interval]
Never smoker						
Used to smoke occasionally	1.012	0.305	0.04	0.967	0.561	1.828
Used to smoke regularly	1.392	0.209	2.2	0.028	1.037	1.868
Current smoker	1.638	0.296	2.74	0.006	1.150	2.333
BMI under 20						
BMI 20-25	0.822	0.278	-0.6	0.561	0.424	1.594
BMI 25-30	0.494	0.169	-2.1	0.039	0.252	0.966
BMI Over 30	0.661	0.225	-1.2	0.224	0.339	1.289
Female sex						
Male sex	1.187	0.150	1.36	0.175	0.926	1.522
GHQ12 Score=0 [best psychosocial health]						
GHQ12 Score=1	1.369	0.238	1.8	0.071	0.973	1.926
GHQ12 Score=2	1.149	0.294	0.54	0.587	0.696	1.898
GHQ12 Score=3	1.332	0.361	1.06	0.291	0.783	2.267
GHQ12 Score=4	2.192	0.613	2.81	0.005	1.267	3.791
GHQ12 Score=5	2.710	0.746	3.62	0	1.580	4.647
GHQ12 Score=6	1.614	0.623	1.24	0.215	0.758	3.439
GHQ12 Score=7	1.686	0.658	1.34	0.181	0.785	3.621
GHQ12 Score=8	1.307	0.642	0.54	0.586	0.499	3.424
GHQ12 Score=9	3.814	1.267	4.03	0	1.989	7.312
GHQ12 Score=10	2.586	1.164	2.11	0.035	1.070	6.250
GHQ12 Score=11	1.700	0.843	1.07	0.285	0.643	4.495
GHQ12 Score=12 [worst psychosocial health]	0.969	0.547	-0.1	0.956	0.321	2.930

Table 11: complete model variable selection

Variable Name	LR Test		Wald Test	
	χ2	Pr	χ2	Pr
Age Group	741.25	0	522.16	0
IMD 2004	27.3	0	27.99	0
Limiting illness	64.46	0	58.13	0
Hypertensive	14.55	0.0001	13.92	0.0002
Ever had high blood pressure	10.99	0.0009	10.92	0.001
On drugs for BP	10.41	0.0013	9.85	0.0017
Smoking	12.33	0.0063	12.24	0.0066
BMI	9.78	0.0205	10.01	0.0185

Variable Name	LR Test		Wald Test	
**Sex**	2.69	0.1012	2.69	0.1008
**GHQ 12 Screen**	18.3	0.1068	20.75	0.0542

1.3.4 Prediction

Another method of assessing performance is to use the regression model to predict the response for each subject. These predictions are called fitted values. The difference between the fitted and the observed values are called residuals. These can then be tabulated against the observed presence of stroke to assess “misclassification” by each model. Prediction by the models is shown in Table 12.

Table 12: model predictions

	Observed	Predicted no stroke	Predicted stroke	Total
Local	No stroke	20,740	0	20,740
	Stroke	493	0	493
Complete, 3 BP variables	No stroke	4,728	1	4,729
	Stroke	183	3	186
Complete, hypertensive	No stroke	10,029	0	10,029
	Stroke	198	0	198
Complete, ever had high BP	No stroke	16,366	1	16,367
	Stroke	302	0	302
Taking drugs prescribed for BP	No stroke	5,772	0	5,772
	Stroke	230	1	231

2 Application of the model

The local model (which includes only those variables that are available at population level i.e. age, sex, ethnicity, smoking status and deprivation score) has been applied to Local Authorities to create prevalence estimates of stroke in those aged 16+ for 2005–2020. Models for PCTs have been created for 2006-2020.

2.1 Assumptions of the modelled estimates

It is assumed that:

- the proportion of smokers, ex-smokers and never-smokers is the same across ethnic groups.
- the proportion of ex-smokers in each age-sex group is the same in all areas.
- the smoking prevalence rates from the model-based estimates of lifestyle behaviours (16) are reliable.
- the prevalence of stroke in those aged under 16 is negligible.

Due to lack of data, it was not possible to treat ex-regular-smokers and ex-occasional-smokers separately. Ex-occasional-smokers are treated as non-smokers.

2.2 Input data

2.2.1 Populations

The stroke prevalence model uses ONS 2005 mid-year population estimates by single year of age and sex.

2.2.1.1 Population projections

In order to calculated estimate prevalence of stroke in the future, population projections were incorporated into the model.

For 2006 and 2007 ONS mid-year population estimates by age and sex for LAs and PCTs were used.

For 2008, 2009 2010, 2015 and 2020 the ONS 2006-based population projections by quinary age band for LAs and PCTs were used.

2.2.2 Smoking status

National (England) proportions of smokers, ex-smokers and current smokers by age and sex are taken from HSfE (2003-2005 pooled). These proportions were then adjusted for each LA/PCT using the synthetic estimates of smoking prevalence for 2003-2005 (16), using the following algorithm.

Local proportion of smokers in age-sex category = national prevalence of smoking in age-sex category * local overall smoking prevalence / national overall smoking prevalence

$$S_{asl} = S_{asn} \times \frac{S_l}{S_n}$$

Local proportion of ex-smokers in age-sex category is not adjusted

$$E_{asl} = E_{asn}$$

Local proportion of never-smokers in age-sex category = 1 – proportion of ex-smokers in age-sex category – local proportion of smokers in age-sex category

$$N_{asl} = 1 - E_{asl} - S_{asl}$$

Where:

S = proportion of population who are smokers
E = proportion of population who are ex-regular-smokers
N = proportion of population who have never smoked
l = local

n = national
as = by age and sex

This approach assumes that the proportion of ex-smokers in each age-sex category is fixed and the number of never-smokers increases as the number of smokers decreases. Regional analysis of the relationship between prevalence of smokers and ex-smokers in the Health Survey for England shows no systematic relationship and therefore it was decided that the ex-smoking rate should not be locally adjusted.

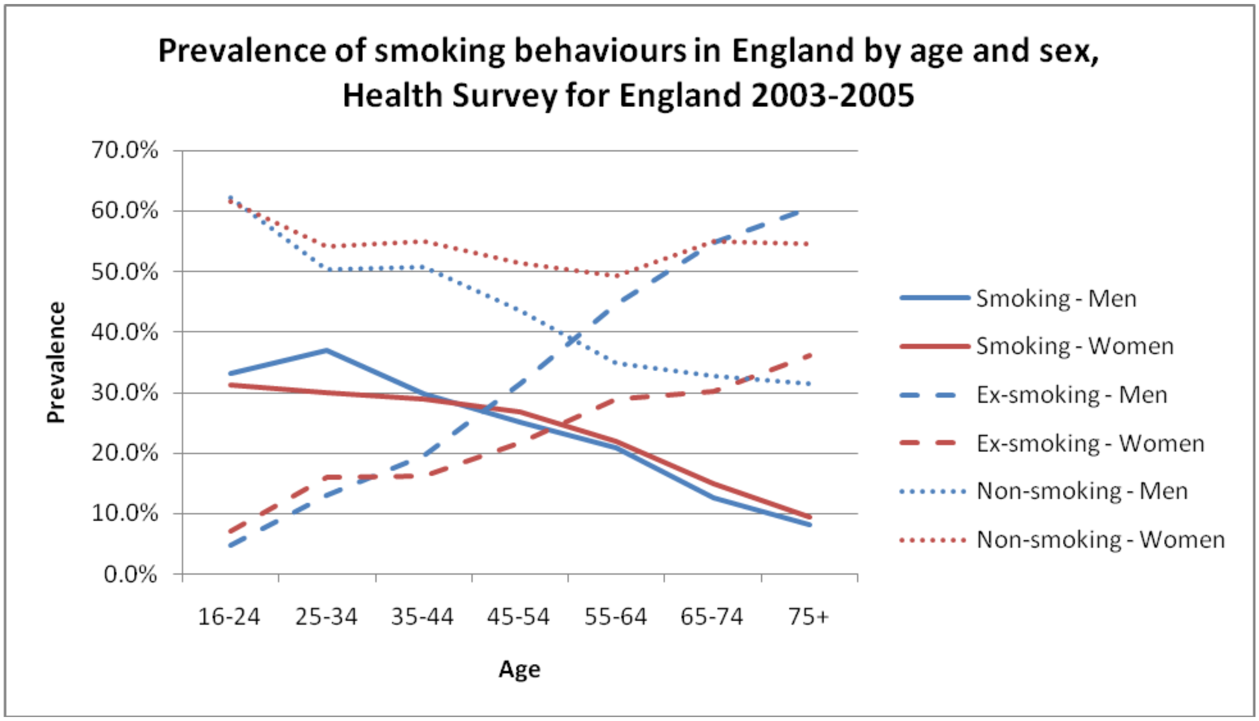

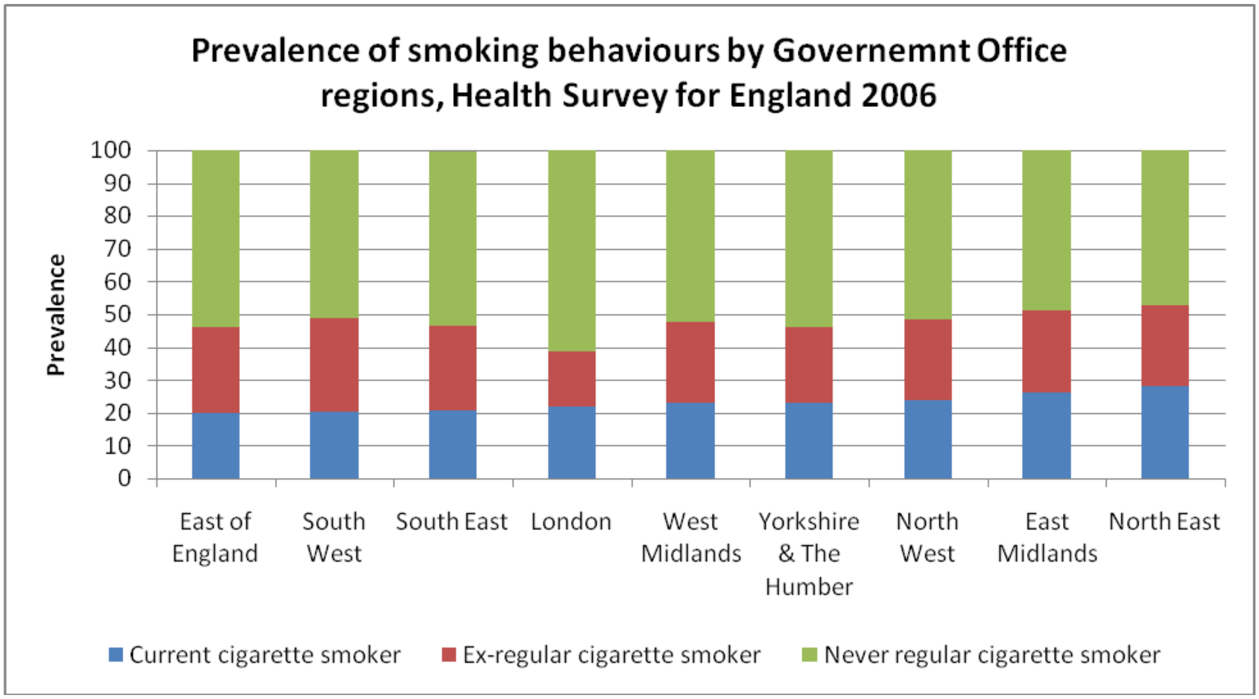

The same smoking prevalence rates are applied across all ethnic categories.

Future changes in smoking prevalence are not taken into account in the stroke prevalence projections. This is because of the uncertainty associated with predictions of smoking prevalence, and the lag time between smoking cessation and improved health. Even if there was a rapid drop in the number of smokers over the next few years, any associated decrease in stroke would not be seen for many years.

2.2.3 Deprivation

Deprivation scores are taken from IMD 2004 (17). Deprivation scores for PCTs were calculated by taking a population weighted average of the scores for each MSOA (which in turn were calculated by taking a weighted average of the IMD2004 scores of each LSOA) within the PCT.

Five deprivation categories are used in the model. Note that these categories are based on quintiles of IMD score at LSOA level. When the cut-offs are applied to larger geographies (LA or PCT) there is not an even distribution across the categories.

2.3 Adjustment for haemorrhagic stroke

The Healthcare Commission requested that the modelled estimates of stroke prevalence be adjusted to remove the contribution of haemorrhagic stroke. The prevalence of haemorrhagic stroke by age used by NICE in the costing template for lipid modification (18) was subtracted from the stroke prevalence in each age band to give the prevalence of non-haemorrhagic stroke.

Table 13: Prevalence of haemorrhagic stroke from NICE costing template for lipid modification (18)

Age	Prevalence of haemorrhagic stroke	
	Male	Female
18-34	0.03%	0.03%
35-44	0.03%	0.03%
45-54	0.03%	0.03%
55-64	0.03%	0.03%
65-74	0.28%	0.26%
75-84	0.69%	0.49%
85+	1.57%	0.67%

3 References

1. Truelsen T, Piechowski J, Bonita R, Mathers C, Bogousslavsky J, Boysen G. Stroke incidence and prevalence in Europe: a review of available data. European Journal of Neurology 2006;13(6):581-98.
2. Feigin VL, Lawes CM, Bennett DA, Anderson CS. Stroke epidemiology: a review of population-based studies of incidence, prevalence, and case-fatality in the late 20th century. The Lancet Neurology 2003 Jan;2(1):43-53.
3. Saposnik G, Del Brutto OH. Stroke in South America: A Systematic Review of Incidence, Prevalence, and Stroke Subtypes. Stroke 2003 Sep 1;34(9):2103-7.
4. Jungehulsing GJ. Prevalence of stroke and stroke symptoms: a population-based survey of 28,090 participants. Neuroepidemiology 2008;30(1):51.
5. Geddes JM, Fear J, Tennant A, Pickering A, Hillman M, Chamberlain MA. Prevalence of self reported stroke in a population in northern England. J Epidemiol Community Health 1996 Apr 1;50(2):140-3.
6. MacDonald BK, Cockerell OC, Sander JWAS, Shorvon SD. The incidence and lifetime prevalence of neurological disorders in a prospective community-based study in the UK. Brain 2000 Apr 1;123(4):665-76.
7. O'Mahony PG, Thomson RG, Dobson R, Rodgers H, James OFW. The prevalence of stroke and associated disability. J Public Health 1999 Jun 1;21(2):166-71.
8. Information Centre for Health & Social Care. Health Survey for England 2004 Volume 1: the health of ethnic minority groups. Information Centre for Health & Social Care 2008 January 31 [cited 2008 Oct 10];1Available from: URL: http://www.ic.nhs.uk/statistics-and-data-collections/health-and-lifestyles/health-survey-for-england/health-survey-for-england-2004:-health-of-ethnic-minorities--full-report
9. Heuschmann PU, Grieve AP, Toschke AM, Rudd AG, Wolfe CDA. Ethnic Group Disparities in 10-Year Trends in Stroke Incidence and Vascular Risk Factors: The South London Stroke Register (SLSR). Stroke 2008 Aug 1;39(8):2204-10.

10. Smeeton NC, Heuschmann PU, Rudd AG, McEvoy AW, Kitchen ND, Sarker SJ, et al. Incidence of Hemorrhagic Stroke in Black Caribbean, Black African, and White Populations: The South London Stroke Register, 1995 2004. Stroke 2007 Dec 1;38(12):3133-8.
11. Tilling K, Sterne JA, Wolfe CD. Estimation of the incidence of stroke using a capture-recapture model including covariates. Int J Epidemiol 2001 Dec 1;30(6):1351-9.
12. Information Centre for Health & Social Care. Health Survey for England 2006 Latest Trends. Information Centre for Health & Social Care 2008 January 31Available from: URL: http://www.ic.nhs.uk/statistics-and-data-collections/healthand-lifestyles-related-surveys/health-survey-for-england/health-survey-for-england-2006-latest-trends
13. Hanley JA, McNeil BJ. The meaning and use of the area under a receiver operating characteristic (ROC) curve. Radiology 1982 Apr 1;143(1):29-36.
14. Hanley JA, McNeil BJ. A method of comparing the areas under receiver operating characteristic curves derived from the same cases. Radiology 1983 Sep 1;148(3):839-43.
15. Zou KH, O'Malley AJ, Mauri L. Receiver-Operating Characteristic Analysis for Evaluating Diagnostic Tests and Predictive Models. Circulation 2007 Feb 6;115(5):654-7.
16. Model based estimates of healthy lifestyle behaviours http://www.ic.nhs.uk/statistics-and-data-collections/population-and-geography/neighbourhood-statistics/neighbourhood-statistics:-model-based-estimates-of-healthy-lifestyle-behaviours-at-pco-level-2003-05
17. IMD 2004 http://www.communities.gov.uk/archived/general-content/communities/indicesofdeprivation/216309/
18. NICE Clinical Guidance 67 - lipid modification: costing template http://www.nice.org.uk/Guidance/CG67/CostTemplate/xls/English
